# Statistical Relationship Between Wastewater Data and Case Notifications for COVID-19 Surveillance in the United States From 2020 to 2023: Bayesian Hierarchical Modeling Approach

**DOI:** 10.2196/68213

**Published:** 2025-05-22

**Authors:** Masahiko Haraguchi, Fayette Klaassen, Ted Cohen, Joshua A Salomon, Nicolas A Menzies

**Affiliations:** 1 Department of Global Health and Population, Harvard T H Chan School of Public Health, 677 Huntington Ave, Boston, MA, 02115, United States, 1 6174321046; 2 Department of Epidemiology of Microbial Diseases and Public Health Modeling Unit, Yale School of Public Health, New Haven, CT, United States; 3 Department of Health Policy, Stanford University, Stanford, CA, United States

**Keywords:** wastewater epidemiology, COVID-19 surveillance, environmental surveillance, SARS-CoV-2, Bayesian modeling, pandemic, wastewater, surveillance, USA, United States of America, Bayesian, hierarchical model, regression model, coronavirus, respiratory, infectious, pulmonary, COVID-19, environment, sewer, emission, sewerage

## Abstract

**Background:**

During the COVID-19 pandemic, several US jurisdictions began to regularly report levels of SARS-CoV-2 in wastewater as a proxy for SARS-CoV-2 incidence. Despite the promise of this approach for improving COVID-19 situational awareness, the degree to which wastewater surveillance data agree with other data has varied, and better evidence is needed to understand the situations in which wastewater surveillance data track closely with traditional surveillance data.

**Objective:**

In this study, we quantified the statistical relationship between wastewater data and traditional case-based surveillance data for multiple jurisdictions.

**Methods:**

We collated data on wastewater SARS-CoV-2 RNA levels and COVID-19 case reports from July 2020 to March 2023 for 107 counties representing a range in terms of geographic location, population size, and urbanicity. For these counties, we used Bayesian hierarchical regression modeling to estimate the statistical relationship between wastewater data and reported cases, allowing for variation in this relationship across counties. We compared different model structural approaches and assessed how the strength of the estimated relationships varied between settings and over time.

**Results:**

Our analyses revealed a strong positive relationship between wastewater data and COVID-19 cases for the majority of locations, with a median correlation coefficient between observed and predicted cases of 0.904 (IQR 0.823‐0.943). In total, 23/107 counties (21.5%) had correlation coefficients below 0.8, and 3/107 (2.8%) had values below 0.6. Across locations, the COVID-19 case rate associated with a given level of wastewater SARS-CoV-2 RNA concentration declined over the study period. Counties with greater population size (*P*<.001) and higher levels of urbanicity (*P*<.001) had stronger concordance between wastewater data and COVID-19 cases. Measures of model fit, and relationships with urbanicity and population size, were robust to sensitivity analyses in which we varied the time period of analysis and the sample of counties used for model fitting.

**Conclusions:**

In a sample of 107 US counties, wastewater surveillance had a close relationship with COVID-19 cases reported for the majority of locations, with these relationships found to be stronger in counties with greater population size and urbanicity. In situations where routine COVID-19 surveillance data are less reliable, wastewater surveillance may be used to track local SARS-CoV-2 incidence trends.

## Introduction

### Background

The practice of using wastewater data to track pathogens has gained significant interest as an innovative method of infectious disease surveillance, with nearly 80% of the US population connected to public wastewater systems [1,2]. During the COVID-19 crisis, local health agencies started to gather and report data on COVID-19 concentrations in wastewater, using the trends in these data as an indirect indicator of SARS-CoV-2 transmission patterns.

Tracking the presence and concentration of pathogens in wastewater, a passive method of environmental surveillance, has been used for several decades to track infectious diseases, such as polio [3], gastroenteritis [4], hepatitis E [5], and acute diarrhea [6], among others. However, the COVID-19 pandemic accelerated interest in this approach worldwide [7], as it offers multiple benefits as a complement to traditional surveillance systems. First, it can serve as an early warning system for SARS-CoV-2 transmission, sensitive to asymptomatic and presymptomatic cases 4-10 days prior to clinical testing signals [8]. Second, it can monitor community-level transmission when implemented at downstream locations such as wastewater treatment plants [9]. The fact that it does not require individual testing circumvents the challenges created by variable supply of and demand for COVID-19 diagnostic testing (as has been observed over the pandemic), and the decline in reporting of test results [10]. Third, wastewater surveillance programs with specific methods can track virus variants in the early stage of their evolution, allowing for early identification of emerging variants [9,11,12]. Finally, it provides an early indicator of epidemiological changes (as compared to hospitalization and death data), so that mitigation and other response measures can be deployed more rapidly. With these advantages, tracking wastewater COVID-19 data is a potentially powerful tool for COVID-19 surveillance.

Despite the potential advantages of SARS-CoV-2 wastewater surveillance, significant challenges remain. Existing studies have generally considered the relationship between reported cases and wastewater metrics at a limited number of locations, such as university dorms [13], nursing homes [14], university campuses [15], and municipalities (eg, Oklahoma City, Oklahoma [16] and Louisville, Kentucky [17]). While these studies provide valuable insights, they often do not fully account for heterogeneities across different geographical locations. Furthermore, the time periods covered by existing studies are limited. For example, Xiao, Wu [18] associated clinical case data with wastewater data within 3 Massachusetts counties from March 2020 through May 2021. Similarly, Weidhaas and Aanderud [19] analyzed 9 weeks of wastewater and COVID-19 case data related to 10 wastewater treatment facilities in Utah. Notably, reported cases themselves are not a perfect indicator for true infection situations, given that they depend on various factors such as testing availability and access as well as the number of asymptomatic infections. As such, while quantifying the relationship between reported cases and wastewater metrics provides valuable insights, it should be noted as the next-best alternative. Studies that compare the performance of COVID-19 wastewater surveillance data across sewersheds over extended time frames remain scarce [20]. Such research is valuable for establishing the statistical basis for real-time trend analysis and describing the conditions under which wastewater surveillance performs well.

### Objectives

This study explored the quantitative relationship between SARS-CoV-2 wastewater surveillance data and reported COVID-19 diagnoses across multiple sewer-sheds over the initial years of the COVID-19 pandemic. Using weekly aggregated wastewater and case report data for 107 US counties over 2020‐2023, we used Bayesian hierarchical modeling to establish the statistical relationship between these 2 data sources, describe changes in these relationships over time and across locations, and identify how the strength of these relationships varied systematically by county characteristics. A visual summary of the study design and findings is provided in Multimedia Appendix 1.

## Methods

### Data Sources

For the study period July 1, 2020, to March 1, 2023, we collated county-level SARS-CoV-2 wastewater surveillance data reported by Biobot Analytics, including 254 counties covering approximately 30% of the US population. These data represent RNA copies per milliliter, normalized by the concentration of pepper mild mottle virus to correct for variability in fecal content, which is influenced by environmental factors such as stormwater [21]. Surveillance data on county-level weekly COVID-19 reported case totals were extracted from the COVID-19 data repository in the Center for Systems Science and Engineering at Johns Hopkins University. This repository aggregates data from a large number of national and subnational US sources, including the CDC, state and country health departments, and nongovernment COVID-19 surveillance projects [22]. Aggregated and harmonized data represent all COVID-19 cases diagnosed and reported to these sources for each reporting period, and therefore will reflect variation in the coverage of COVID-19 testing and completeness of reporting over time and between locations. Both the Biobot Analytics data and the COVID-19 data repository at Johns Hopkins University consist of aggregated and deidentified data. No individual-level or personally identifiable information was used in the analysis.

**Figure 1.**
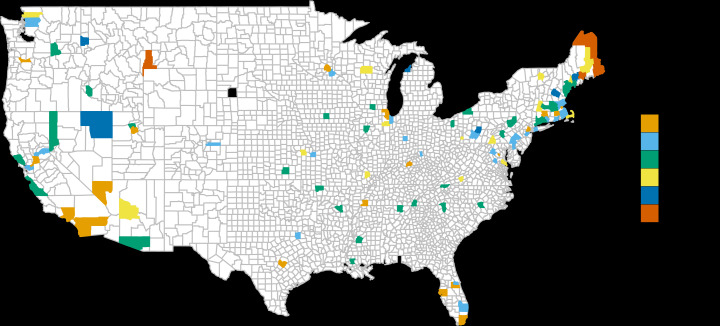
This shows geographic location and National Center for Health Statistics urbanicity category for the 107 counties included in the analysis. NCHS: National Center for Health Statistics.

Due to varied implementation of wastewater surveillance operations, wastewater data were not available for all county-weeks. We restricted the analysis to counties with a minimum of 50 weeks of available wastewater data during the study period, which resulted in 107 counties being included, covering a range of geographic areas within the United States, and with periods of data incompleteness for the majority of counties. We assessed the correlation between wastewater metrics and case totals with different time lags (0 wk, 1 wk, 2 wk, etc), and found that a 0 week time lag has the highest correlation. Therefore, we adopted this for the analysis. We grouped counties into 6 ordinal urbanicity categories as defined by the National Center for Health Statistics (NCHS). Categories ranged from NCHS category 1 (large central metro) as the most urban to NCHS category 6 (noncore) as the most rural. Figure 1 shows the geographic distribution of the 107 counties included in the analysis, coded by NCHS urbanicity category.

### Hierarchical Regression Models

Using the wastewater and COVID-19 case data, we constructed hierarchical Bayesian regression models to capture key features of each data source, allowing for differences in the estimated relationship between these data over time and between modeled counties. Accounting for temporal variations is vital as the dynamics of the pandemic changed over time due to factors such as the emergence of new variants, public health interventions, vaccination rollouts, and population immunity. Also, allowing for spatial variations accounts for local differences, such as vaccination coverage, socioeconomic and demographic characteristics, and variation in COVID-19 testing and reporting practices. As all of them influence the relationship between wastewater metrics and reported cases, a model that can account for the complex and evolving nature of the pandemic is critical. Namely, we modelled the weekly COVID-19 case reports for each county using a negative binomial likelihood, allowing for over-dispersion in these data:


(1) $$Y_{it}∼NegBin\left(\mu_{it},\varphi \right)$$

(2) $$\mu_{it}=exp\left(\alpha_{t}+\beta_{0i}+\beta_{1i}*X_{it}\right)*n_{i}$$

In Equation 1, $Y_{it}$ represents reported COVID-19 cases for county i and week t. We used an alternative parameterization of the negative binomial in which $\mu_{it}$ parameterizes the mean of the likelihood and φ parameterizes the extra-Poisson variation. The mean was specified as a function of $\alpha_{t}$, a time-varying coefficient given a random-walk prior; $\beta_{0i}$, a county-specific intercept; and $\beta_{1i},$a county-specific slope term applied to $X_{it}$, the demeaned wastewater COVID-19 concentration value for each county and week (Equation 2). Both $\beta_{0i}$ and $\beta_{1i}$ were specified as random effects to pool information across counties. $n_{i}$ represented the population of each county, as reported by the US Census Bureau. We fit this model to the COVID-19 case and wastewater data using the RStan package in R (Stan Development Team) [23]. The prior distributions used in this model, including weakly informative priors for fixed effects and random effects coefficients, were selected based on their widespread use in Bayesian modeling. These priors serve to regularize parameter estimates without excessively constraining the model [24]. Sensitivity checks confirmed that the priors do not strongly influence the posterior estimates. Additional details on the specification of this regression model, including prior distributions, are provided in the Multimedia Appendix 2.

### Analysis of Fitted Models

We used several approaches to assess model fit. First, we visually compared the COVID-19 case time series for each county to the fitted values from the regression model. Second, we calculated a quantitative measure of model fit: the median absolute deviation (MAD). We estimated this value for each county and used them to describe the overall level of model fit and how this varied across counties. We used the MAD to investigate whether the strength of estimated relationships differed systematically as a function of county characteristics (urbanicity and population size), examining these relationships visually and via univariable and multivariable regression models. Finally, we calculated the correlation coefficient between modeled values and raw case totals as a simple summary measure of model fit.

### Coefficient Estimates

We used the fitted values of $\alpha_{t}$ (the temporal trend in the regression model) to understand how the relationship between wastewater and COVID-19 case totals varied over the study period (from July 2020 to March 2023). We used the fitted values of $\beta_{0i}$ and $\beta_{1i}$ to understand how the relationship between wastewater concentration and COVID-19 case totals varied within each county.

### Sensitivity Analyses

We conducted 2 sets of sensitivity analyses to evaluate the robustness of the model results. First, we divided the dataset into 3 equal 11-month time periods (early, mid, and late pandemic). We refit the model for each of these time periods, and compared the goodness-of-fit metrics (MAD) and analyses of county characteristics (urbanicity and population size) from these 3 models to the results of the main analysis. Second, we identified counties with high data completeness (at least 100 wk of data out of a total of 140 wk in the study period) and refit the model using only these counties, then compared these results to those of the main analysis.

## Results

### Fitted Relationship Between COVID-19 Cases and Wastewater Concentration

**Figure 2.**
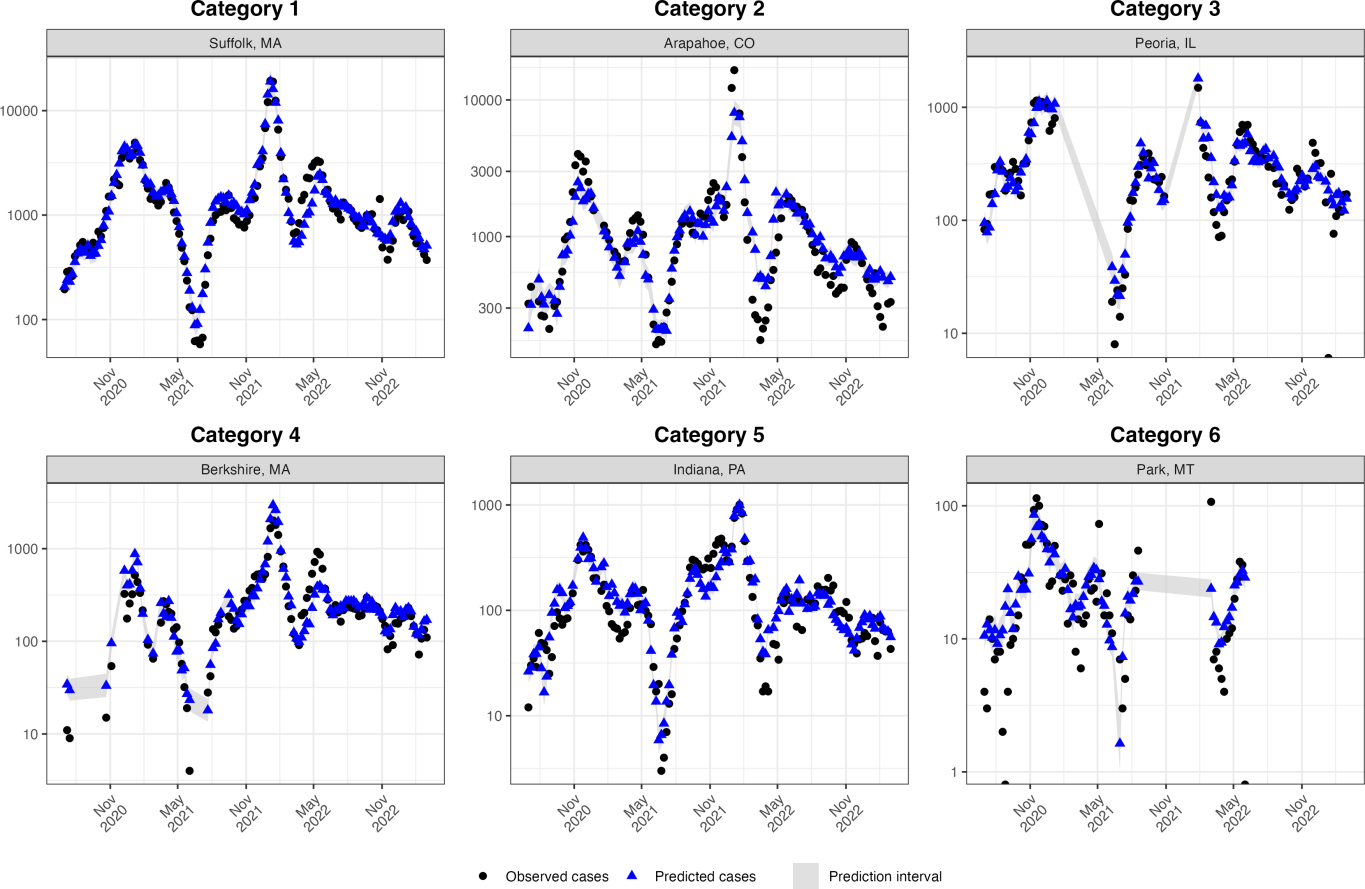
Comparison of observed and predicted COVID-19 case counts for a select group of counties within each urban-rural category as defined by the National Center for Health Statistics (NCHS). Category 1 represents the most urban counties, while Category 6 denotes the most rural ones.

For the majority of modeled locations, we estimated a relationship between wastewater concentration and weekly COVID-19 cases, indicating that wastewater concentration serves as a useful predictor of case trends. Figure 2 shows the temporal trend in reported COVID-19 cases and fitted model estimates for each of the 6 example countries, representing a range in terms of urbanicity and population size, which are often correlated but not identical. For each of these example countries the fitted model values (blue symbols) follow the empirical case data (black symbols) closely, with occasional deviations (eg, late 2021 - early 2022 estimates for Arapahoe County, CO). Figures for other counties are available in Figure S1 in Multimedia Appendix 2. We also calculated correlation coefficients comparing observed and predicted values of COVID-19 case counts. Across counties, the median of these correlation coefficients was 0.904 (IQR 0.823-0.943). In total, 23/107 counties (21.5% of the sample) had correlation coefficients below 0.8, and 3 had values below 0.6.

### Systematic Differences in Model Fit Across Counties

**Figure 3.**
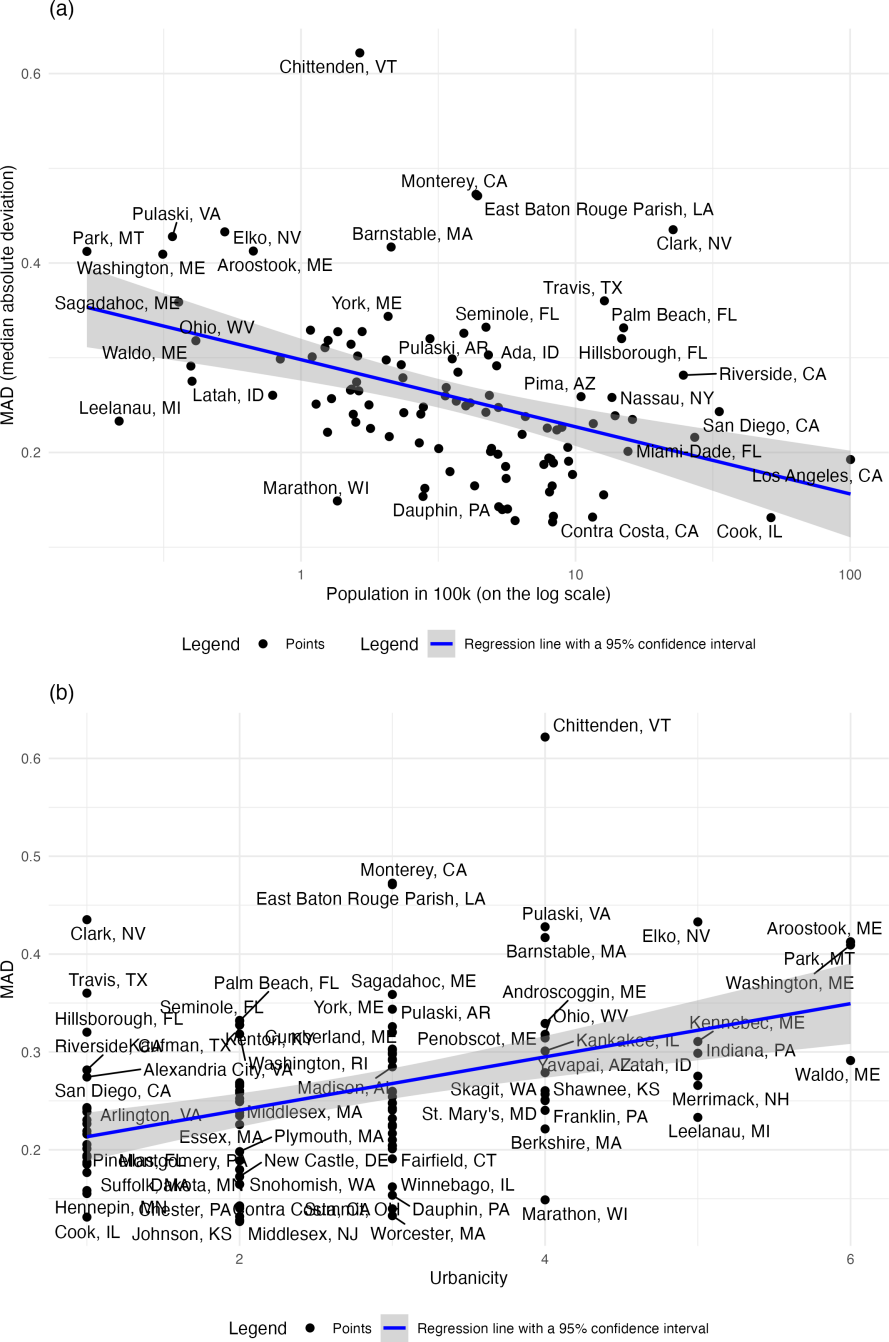
(A) How the quality of model fit (MAD) varies with county population size. (B) How the quality of model fit (MAD) varies with country urbanicity category. Category 1 represents the most urban counties, while Category 6 denotes the most rural ones.

To further evaluate how the model performed in each county, we calculated the MAD for which smaller values indicate better model fit. For MAD, the median value was 0.259 (IQR 0.201-0.301). When we compared MAD to country population size (Figure 3A), we found that model fit was better for counties with higher population numbers (*P*<.001).

Furthermore, we found that MAD was associated with urbanicity (Figure 3B), with more urban counties (Categories 1, 2, and 3) having lower MAD (*P*<.001) and therefore better model fit. More rural counties had poorer model fits (higher values of MAD), with the exception of Chittenden VT.

When we fit a multivariable regression model including both logged population and urbanicity categories as predictors, we found both coefficients to have the same sign as in the univariate analyses but were no longer significant (population size: *P*=.09; urbanicity: *P*=.13).

### Time Trends

**Figure 4.**
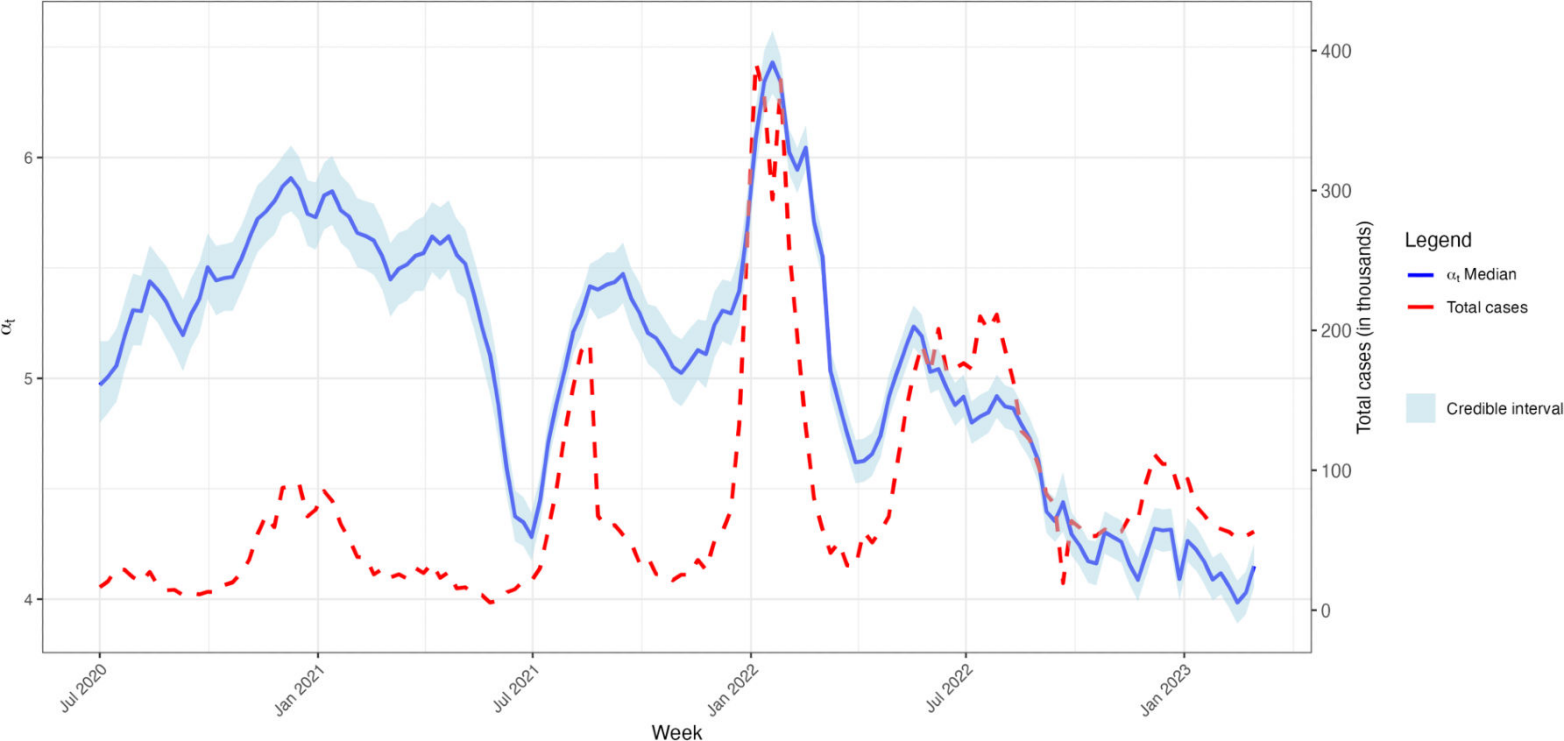
Time series of the time-varying coefficient ($\alpha_{t}$) alongside the aggregated case counts across counties from July 2020 to February 2023.

Figure 4 shows changes in the estimated relationship between wastewater concentration and COVID-19 case reports over the study period, quantified as the level of logged COVID-19 case totals consistent with a given wastewater concentration, shown in blue. Notably, the fluctuations in this relationship are closely associated with the significant US waves at the end of 2020 to the beginning of 2021 (Alpha wave), the summer of 2021 (Delta wave), and the beginning of 2022 (Omicron wave). During these periods, the ratio of COVID-19 cases to wastewater concentration is relatively high as compared to the months before and afterwards. After early 2022, the estimated trend shows a progressive decline.

### Differences Across Counties

**Figure 5.**
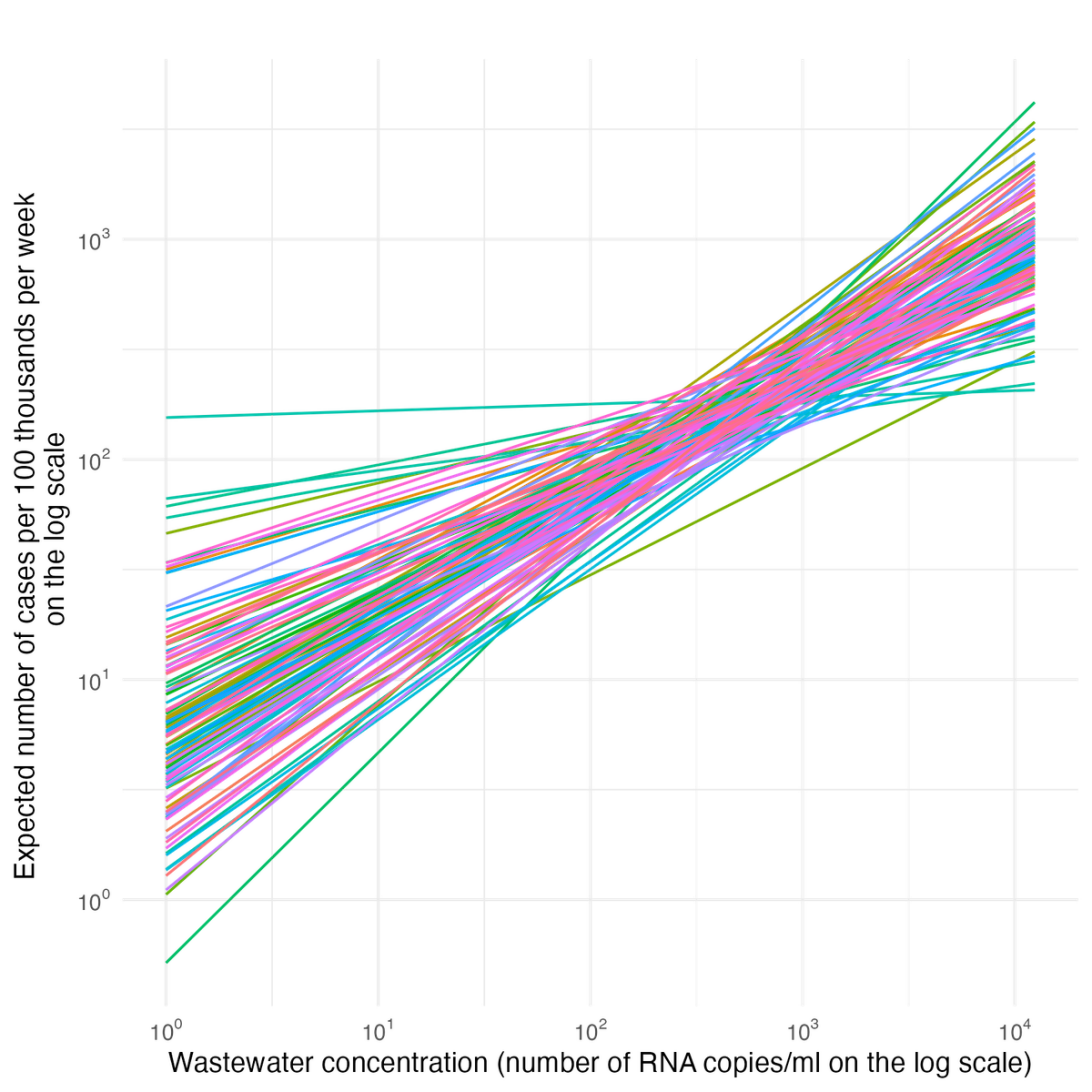
Estimated relationship between wastewater concentration and mean weekly cases per 100 thousand people for each county.

Figure 5 shows fitted estimates for how changes in wastewater concentration values are associated with changes in COVID-19 cases for each county. As expected, the slopes shown in Figure 5 are positive (indicating that an increase in wastewater concentration was associated with an increase in reported cases), and relatively consistent across counties. In all cases, the slope of these lines was estimated to be less than 1 (median 0.551, IQR 0.447-0.632), indicating that the fitted relationship between wastewater concentration and COVID-19 case totals is less than proportional (eg, a 50% increase in wastewater concentration is associated with a <50% increase in case totals).

### Results of Sensitivity Analysis

In the first sensitivity analysis, we re-estimated key results after fitting the study model to each of three equal 11-month time periods subdividing the overall study period into early, mid, and late pandemic. Estimated median MAD values from these sensitivity analyses were 0.19 (IQR 0.15‐0.26) for the early-pandemic period (July 2020-May 2021), 0.29 (IQR 0.22‐0.38) for the mid-pandemic period (June 2020-April 2022), and 0.2 (IQR 0.14‐0.26) for the late-pandemic period April 2022-March 2023. Overall, their results are similar to the MAD estimates from the main analysis (median 0.26, IQR 0.2-0.3), and show the general quality of model fit was generally consistent over the study period. Similarly, estimated trends in MAD as a function of population size and urbanicity matched those of the main analysis (MAD lower with larger population size and greater urbanicity), with results shown in Figures S2-S7 in Multimedia Appendix 2.

In the second sensitivity analysis, we re-estimated results after fitting the study model to countries with high data completeness (19 counties included). From this sensitivity analysis, we found a median MAD value of 0.22 (IQR 0.2‐0.26) comparable to the value of 0.26 (IQR 0.2-0.3) from the main analysis. Estimated trends in MAD as a function of population size and urbanicity matched those of the main analysis (Figures S8 and S9 in Multimedia Appendix 2).

## Discussion

### Principal Findings

Wastewater data have been used extensively during the SARS-CoV-2 pandemic to monitor disease trends and provide early evidence of rising community transmission. However, there is limited information on the statistical relationship between wastewater metrics and reported COVID-19 cases, and how this relationship varies over time and across jurisdictions [20]. This knowledge is valuable for making decisions about how best to use wastewater data, and to understand the settings in which these data provide accurate information about COVID-19 case trends. In this study, we modeled the relationship between wastewater metrics and clinical cases at the county level in the United States from July 2020 to March 2023.

The results of our analysis show that models fit to wastewater data are better able to predict case counts in urban counties (based on NCHS categorization) as compared to more rural counties. This may be due to rural areas having lower levels of connection to centralized sewage systems, the source of wastewater surveillance data [10,25]. We also noted a reduction in model performance among counties with smaller population sizes. This is consistent with other studies that have reported wastewater surveillance to have limited sensitivity as an early warning indicator in smaller geospatial scales [17,26]. We also estimated differences in the quality of model fit that were not explained by urbanicity and population size—these may relate to local differences in the coverage of wastewater surveillance, the processing of wastewater samples, or the quality of COVID-19 case reporting.

In addition to intercounty differences, the results also revealed fluctuations in the relationship between wastewater concentration and COVID-19 case totals over the course of the pandemic. Several factors could account for these temporal trends. First, viral shedding patterns among individuals developing COVID-19 in recent years likely differ from those developing the disease in the early stage of the pandemic, as immunity in the population through previous infections and vaccination increased significantly throughout the pandemic [27]. Second, the transition between different dominant variants may have also influenced the dynamics of discharged RNA copies in human waste. For example, the viral and antibody dynamics are distinct between omicron and delta variants [28]. Also, as mutations may affect the quantification of SARS-CoV-2 concentration in wastewater, such viral changes may need to be accounted for in estimating the relationship between wastewater levels and case notifications [29] . Finally, case reporting systems have changed over time, influencing the ratio of reported and unreported cases [30,31]. In particular, the declining trend over the final year of the time series (ie, a declining number of reported COVID-19 cases for a given wastewater level) likely relates to changes in COVID-19 testing and reporting practices, with a progressively smaller fraction of COVID-19 cases diagnosed and reported to public health authorities.

### Future Directions

Designing effective wastewater surveillance systems requires trade-offs among cost-effectiveness, speed, and local feasibility [32]. Most current sequencing is implemented with hundreds to thousands of samples in parallel with expensive machinery and intensive investment in human resources [32]. This implies that counties with fewer resources may find it difficult to finance and support the required laboratory infrastructure and human resources.

While the quality of model fit was generally good, our analyses revealed substantial variation in the utility of wastewater surveillance across counties. It is also important to note that reported case counts, which we used as a proxy for infection trends, are an imperfect measure of true incidence [33], as they will reflect variation in COVID-19 diagnosis and reporting practices over time and across locations. Ideally, a population-based survey, such as the United Kingdom’s Office for National Statistics’ COVID-19 Infection Survey, would provide more accurate information for assessing the predictive performance of wastewater surveillance. However, without such data in the United States, we rely on case reports as the best available data.

The pepper mild mottle virus normalization method applied to the Biobot wastewater data corrects for variability in fecal content due to environmental factors such as stormwater. However, it may not fully account for seasonal changes or weather-related fluctuations that could influence the observed RNA concentrations. While this method is widely accepted in the field, future research could explore advanced normalization techniques to further refine nationwide wastewater surveillance models.

Further investigation, validation, and standardized data collection frameworks are required to better understand the relationship between wastewater and epidemiological data. The incorporation of next-generation sequencing and automation of wastewater data collection processes could enhance the effectiveness of wastewater surveillance. [34,35].

### Conclusions

The SARS-CoV-2 pandemic made it clear that traditional event-based surveillance systems have critical deficiencies for providing prompt and valid information about the local epidemiological situation. Wastewater surveillance may provide health agencies with another early detection and effective surveillance tool, unaffected by several of the deficiencies of traditional surveillance data. As of March 2024, more than 1300 locations in the United States and over 72 countries globally conducted wastewater surveillance [36,37]. When implemented effectively, these data can provide a comprehensive picture of SARS-CoV-2 transmission, capturing asymptomatic and nontested infections. Our study demonstrates that analyzing wastewater metrics across multiple jurisdictions can establish the relationship between wastewater and potential cases, and how these differ across locations and over time. However, the missing data in wastewater and uncertainty in case data require future efforts to make the relationship between them more established. Efforts to collect wastewater data in a more standardized manner should be enhanced further to fully realize their potential.

## Supplementary material

10.2196/68213 Multimedia Appendix 1 Visual abstract.

10.2196/68213 Multimedia Appendix 2 Supplementary tables and figures on the prior distributions used in the Bayesian model, comparisons of observed and predicted COVID-19 cases across all studied counties, and sensitivity analyses of how MAD varies with population size and urbanicity. MAD: median absolute deviation.

## Abbreviations

CDC: Centers for Disease Control and Prevention
MAD: median absolute deviation
NCHS: National Center for Health Statistics
