# Supplementary figures and images for: Statistical Relationship Between Wastewater Data and Case Notifications for COVID-19 Surveillance in the United States From 2020 to 2023: Bayesian Hierarchical Modeling Approach
Source: JMIR Public Health Surveill. 2025 May 22;11:e68213. doi: 10.2196/68213 (PMC12121544; doi:10.2196/68213)

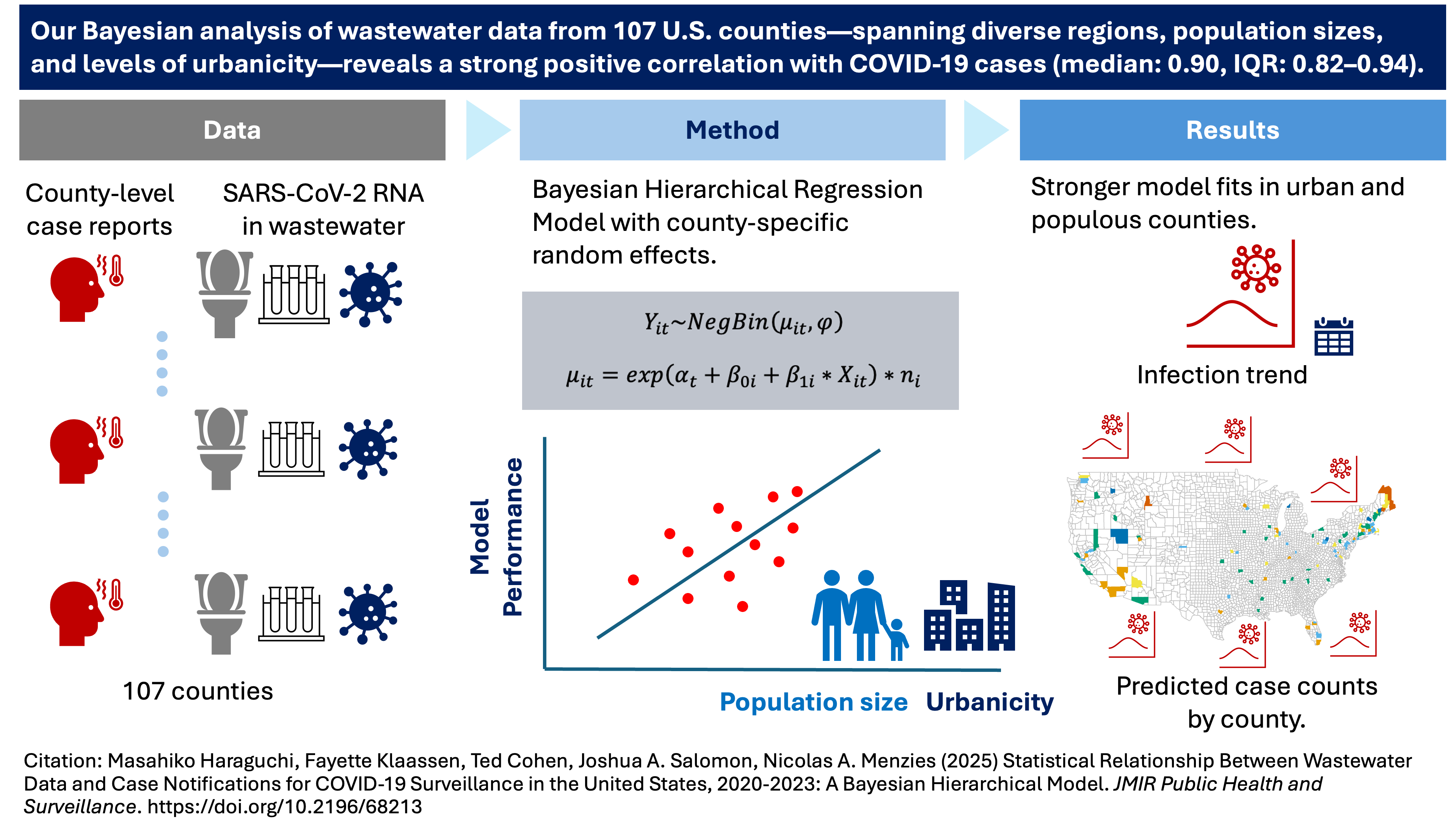

Supplement: Multimedia Appendix 1 [file publichealth-v11-e68213-s001.png]
