# Supplementary material for: Statistical Relationship Between Wastewater Data and Case Notifications for COVID-19 Surveillance in the United States From 2020 to 2023: Bayesian Hierarchical Modeling Approach
Source: JMIR Public Health Surveill. 2025 May 22;11:e68213. doi: 10.2196/68213 (PMC12121544; doi:10.2196/68213)
Supplement: Multimedia Appendix 2 [file publichealth-v11-e68213-s002.docx]

Appendix

# Prior distribution

Prior distribution on intercepts and slopes for concentration. We use the following priors summarized in Table S-1

Table S-1: List of prior distributions

| Model Parameter | Distribution |
| --- | --- |
| Intercept term that includes a random walk effect. (β_00_) | Normal (0, 10) |
| Coefficient of the random effect term for the intercept of the equation (β_0_re_) | Normal(0, σ_0_re_) |
| Standard deviation of the random effect term β_0_re_ (σ_0_re_ ) | Cauchy(0, 5) |
| A fixed effect of wastewater concentration (β_10_) | Normal(0, 10) |
| Random effect of wastewater concentration (β_1_re_) | Normal(0, σ_0_re_); |
| Standard deviation parameter of the random effect term β_1_re_ (σ_0_re_ ) | Cauchy(0, 5) |
| White noise (or random fluctuation) term for the random walk model (β_00_fd_) | Normal(0, σ_fd_) |
| Standard deviation for the random walk’s first differences (σ_fd_) | Cauchy(0, 5) |
| Dispersion parameter for a negative binomial distribution  ($\frac{1}{\sqrt{\varphi}}$) | Normal (0, 1) |

# Figures

## Observed and predicted cases for all counties analyzed.

These are the observed and predicted case counts for all 107 counties included in the analysis.
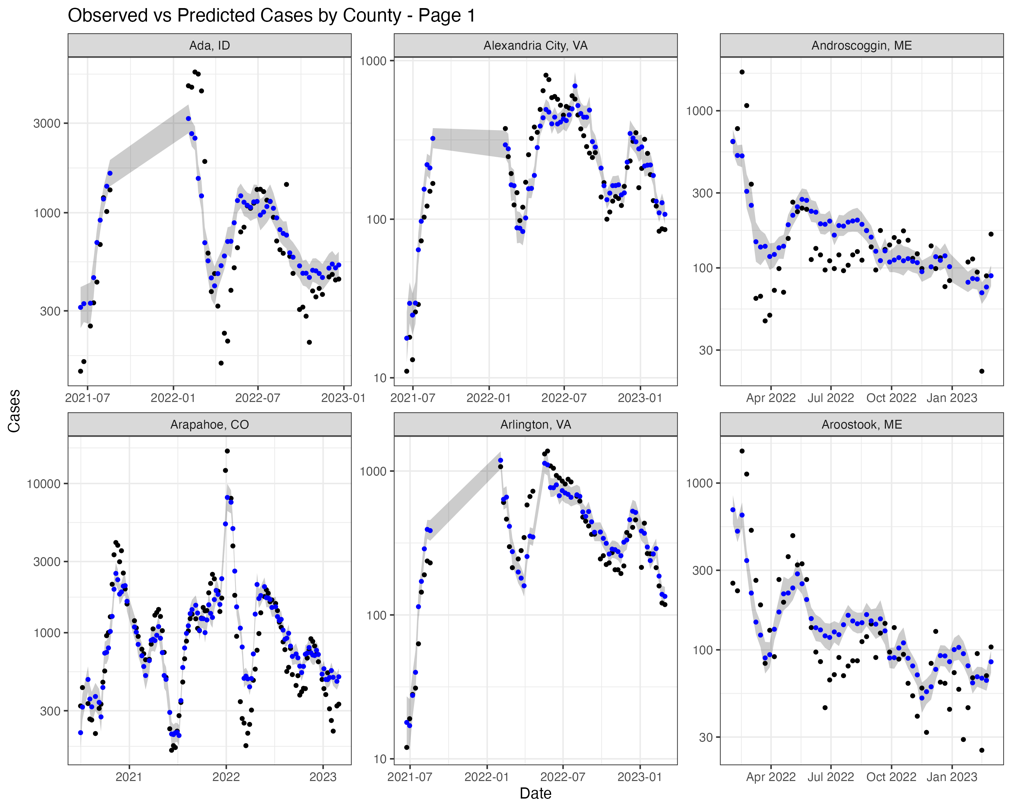


#
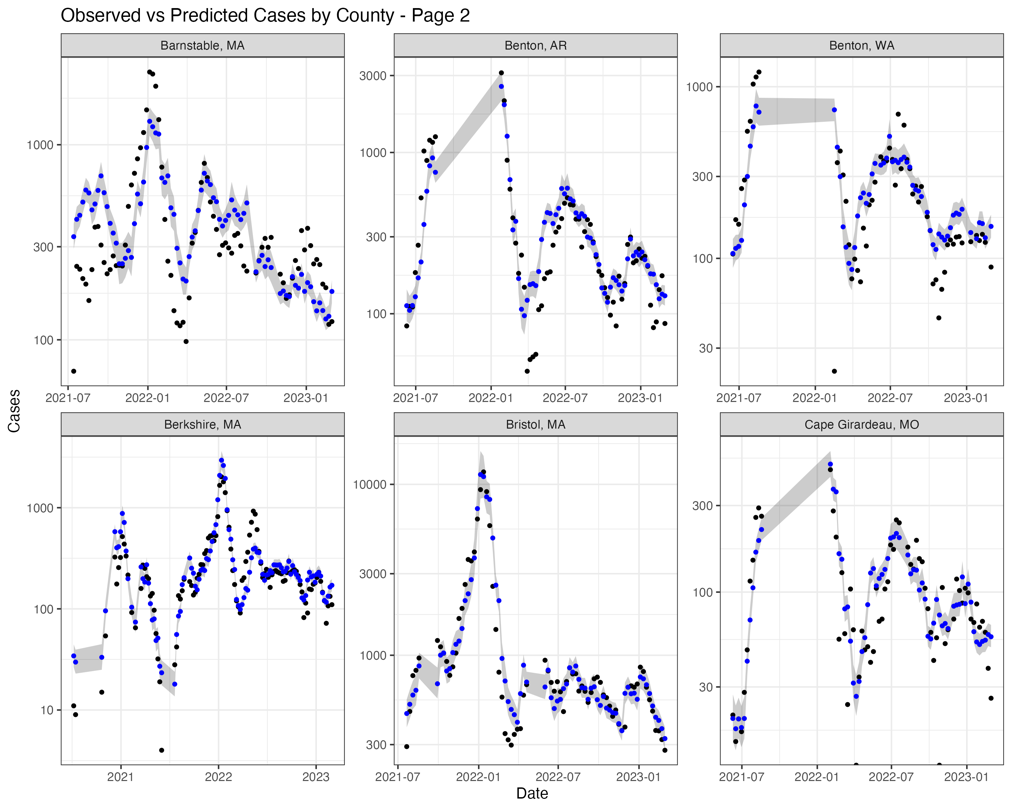

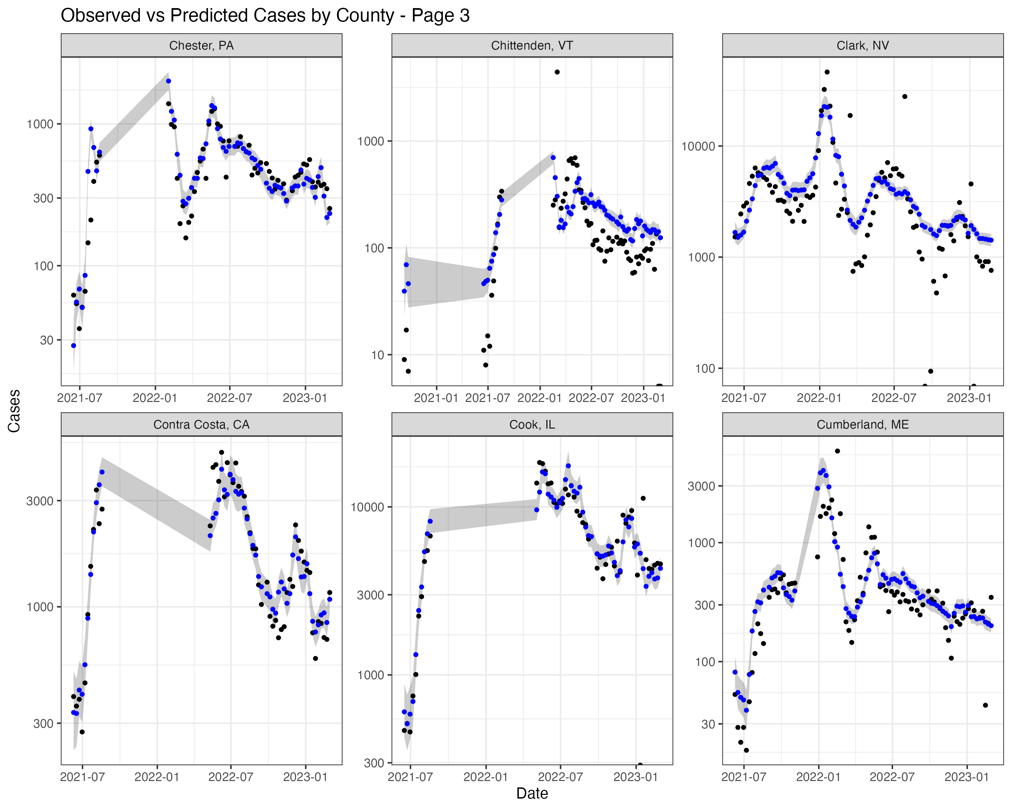

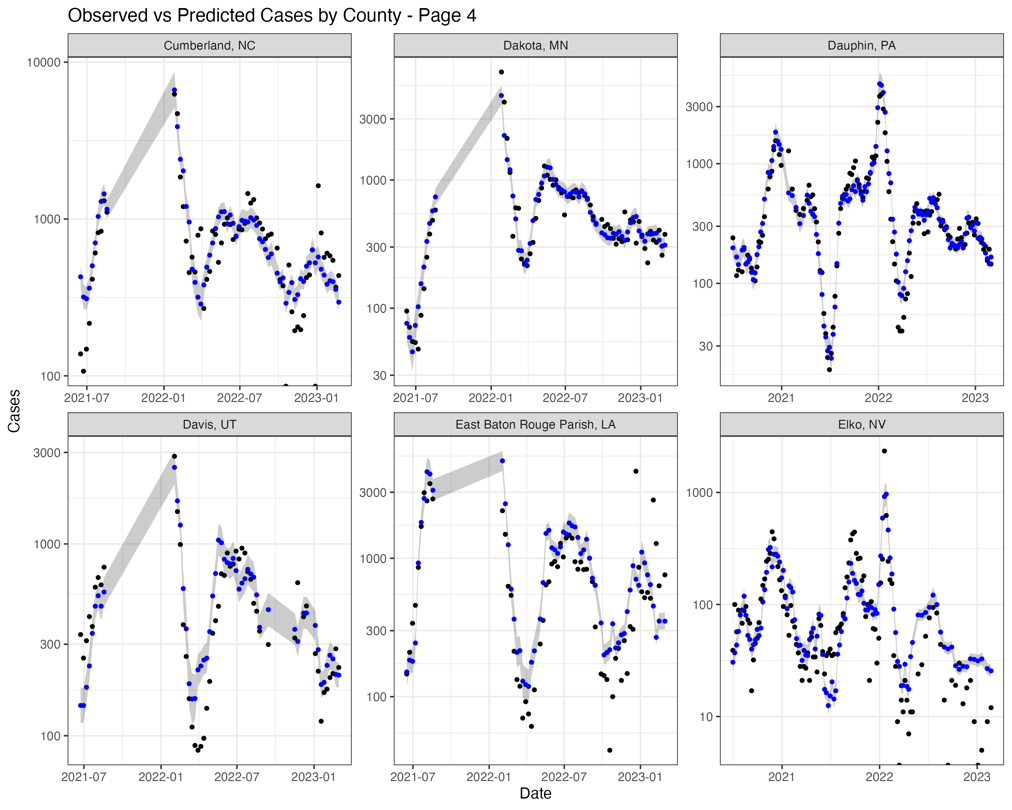

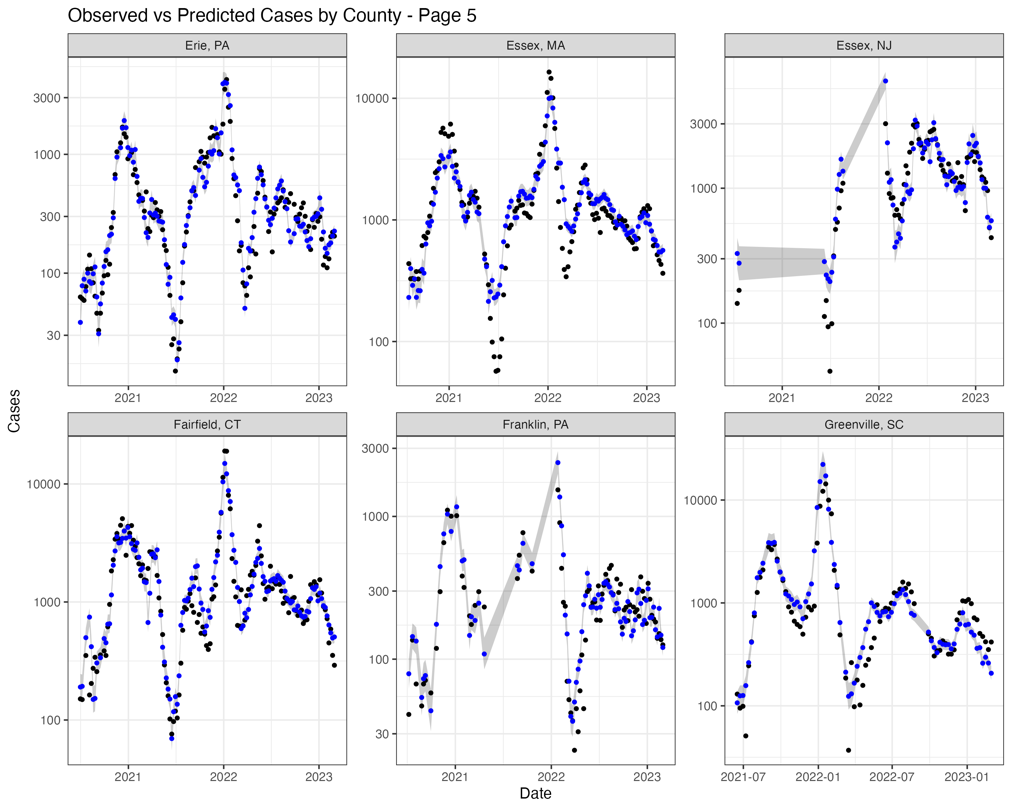

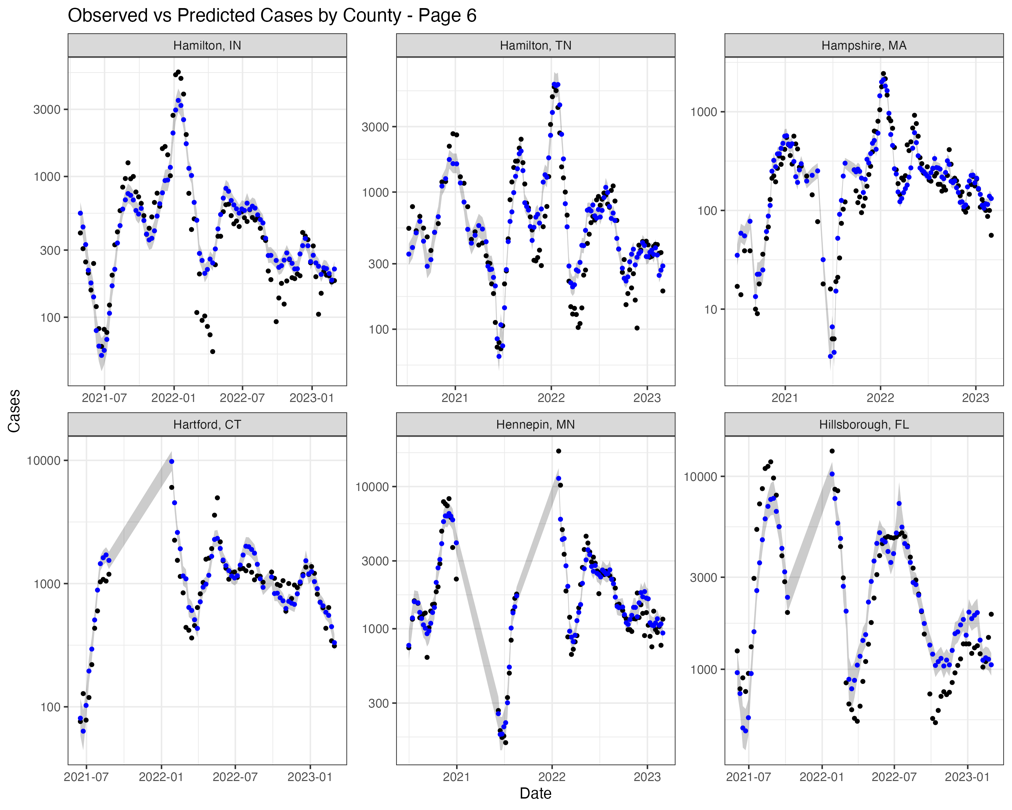

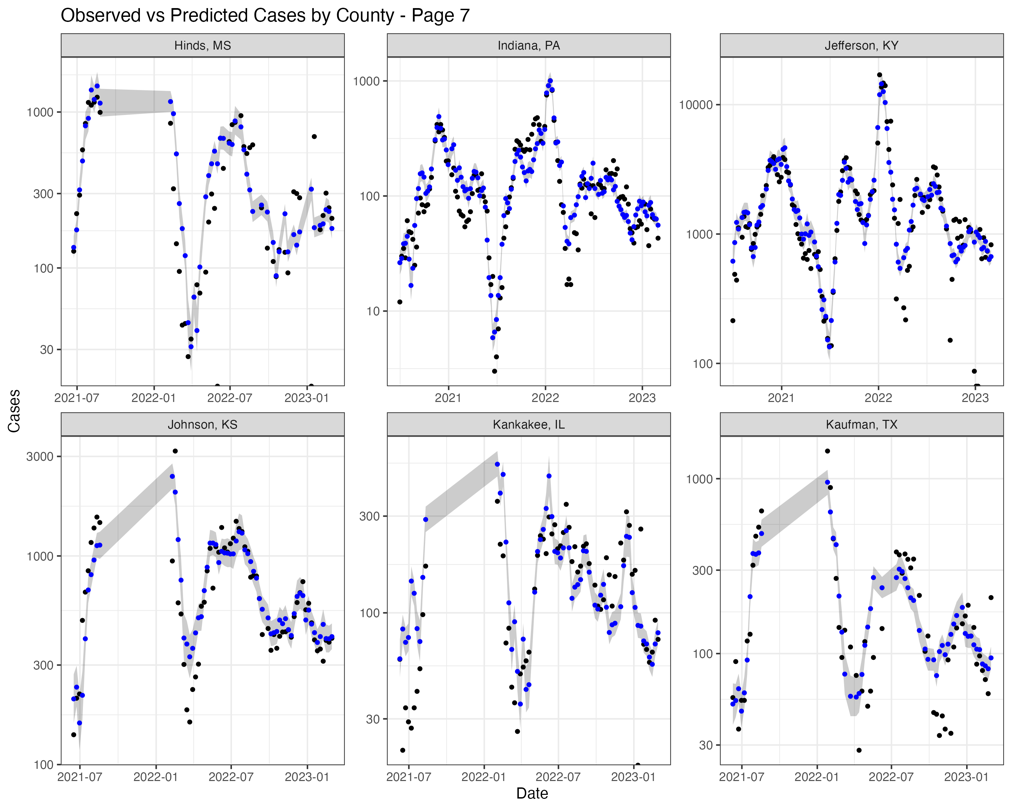

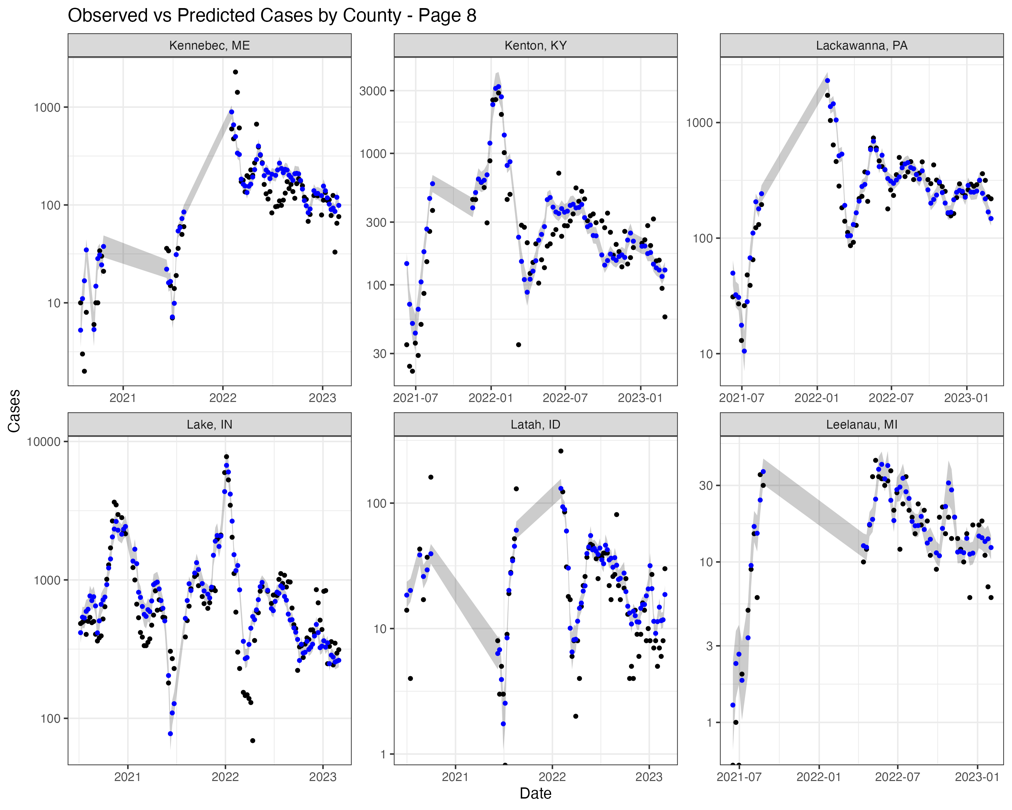

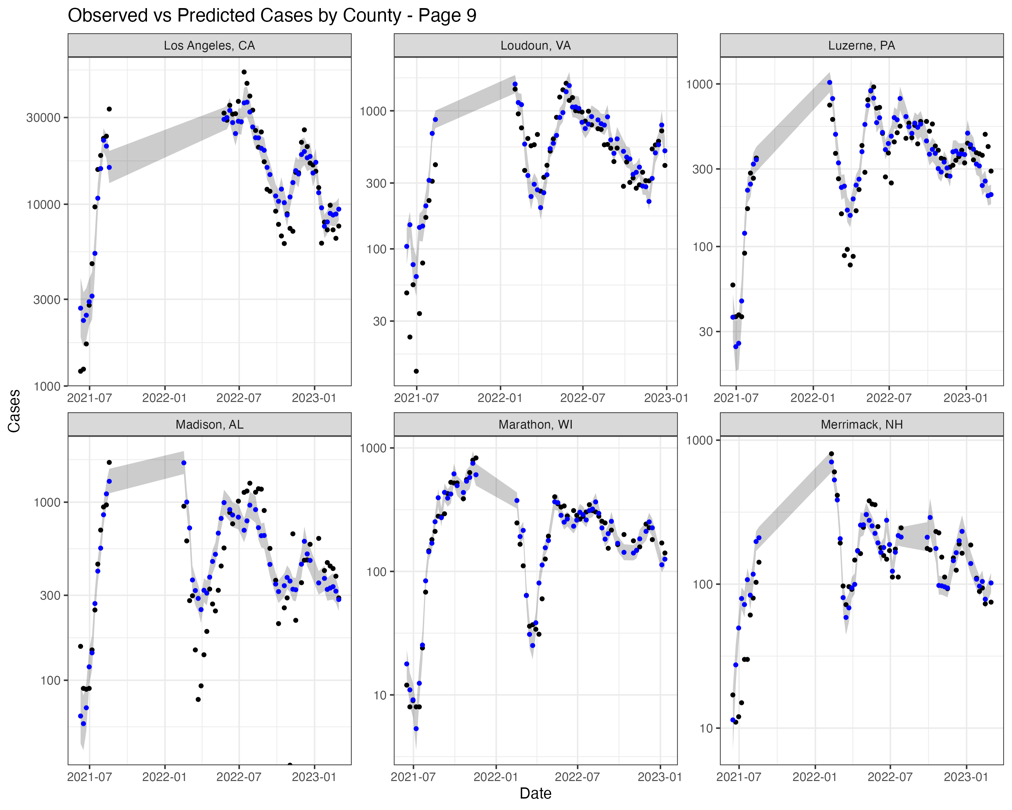

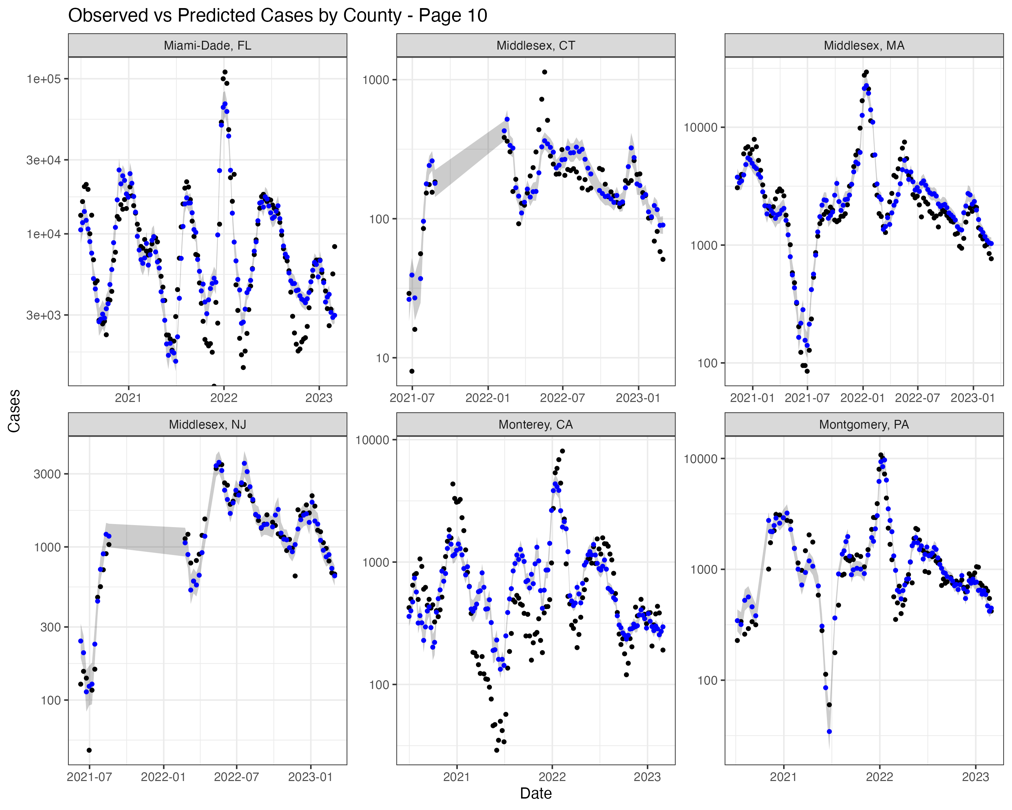

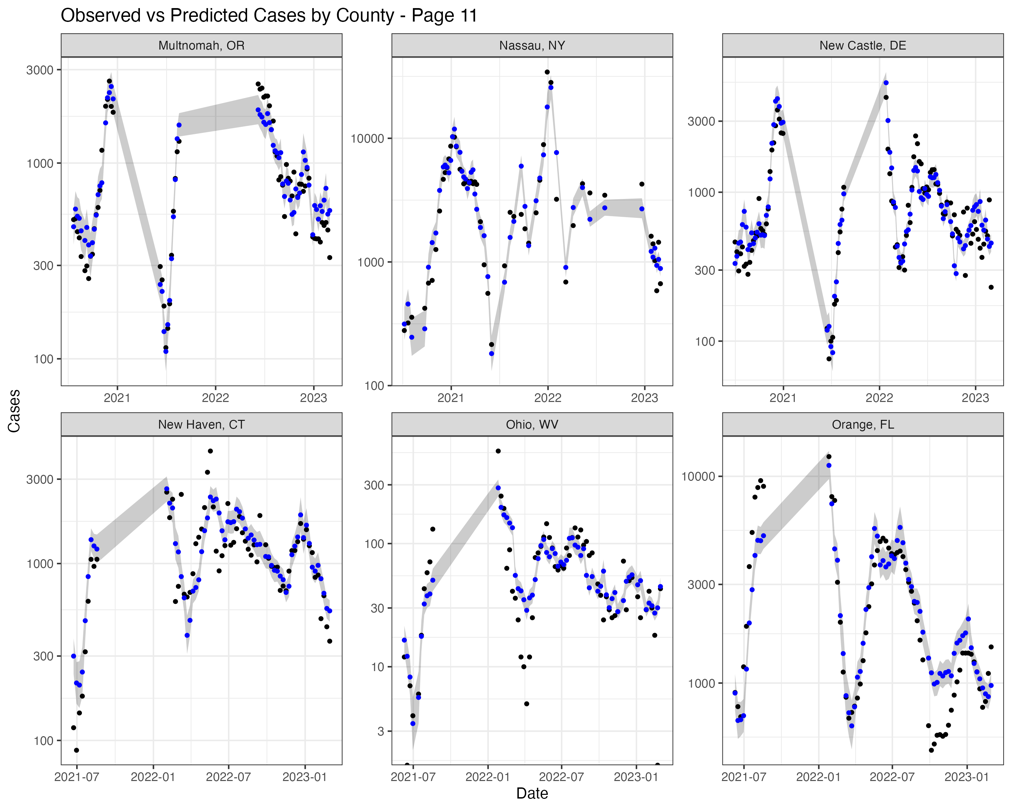

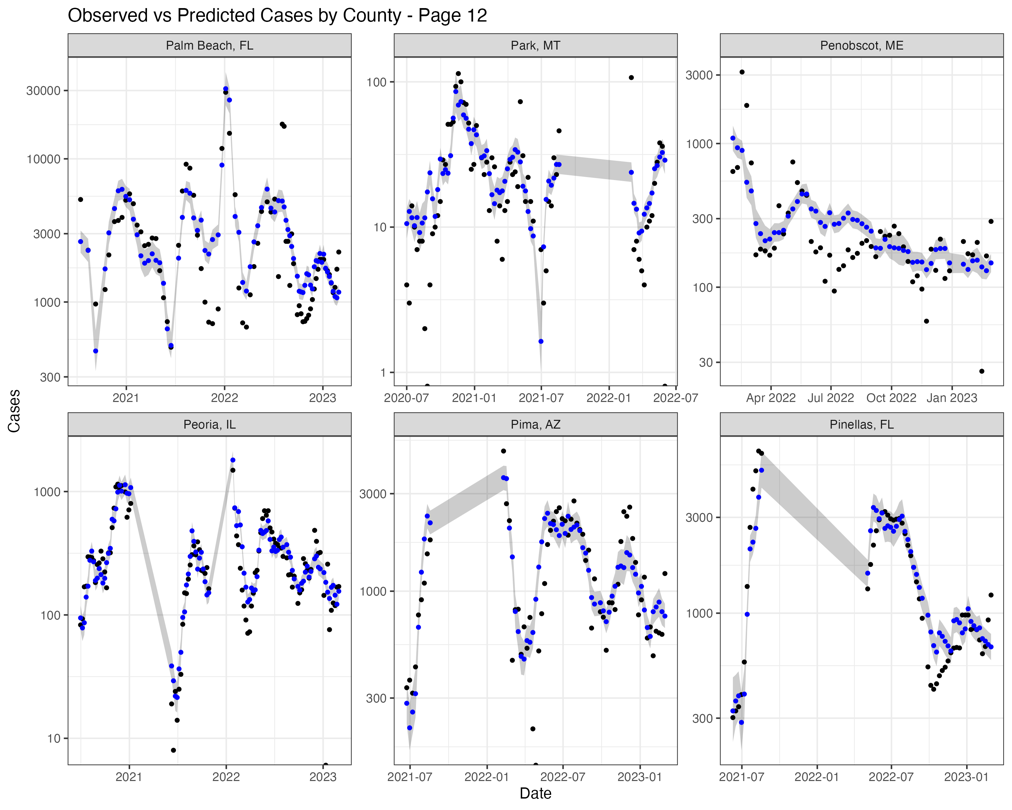

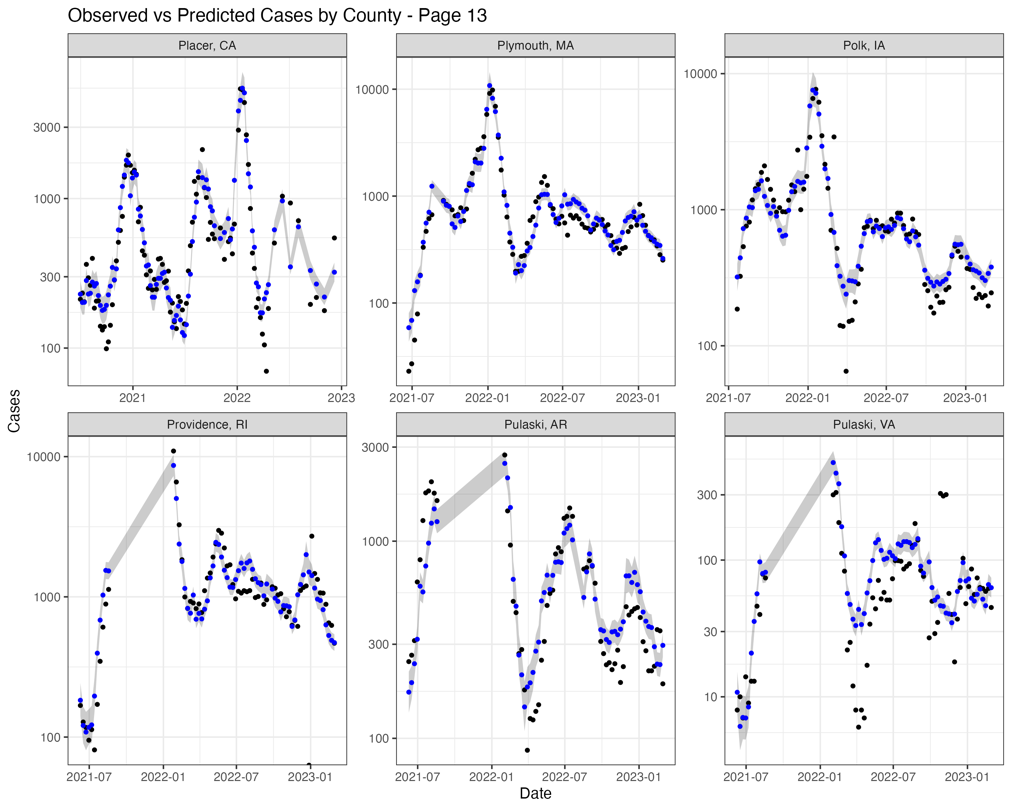

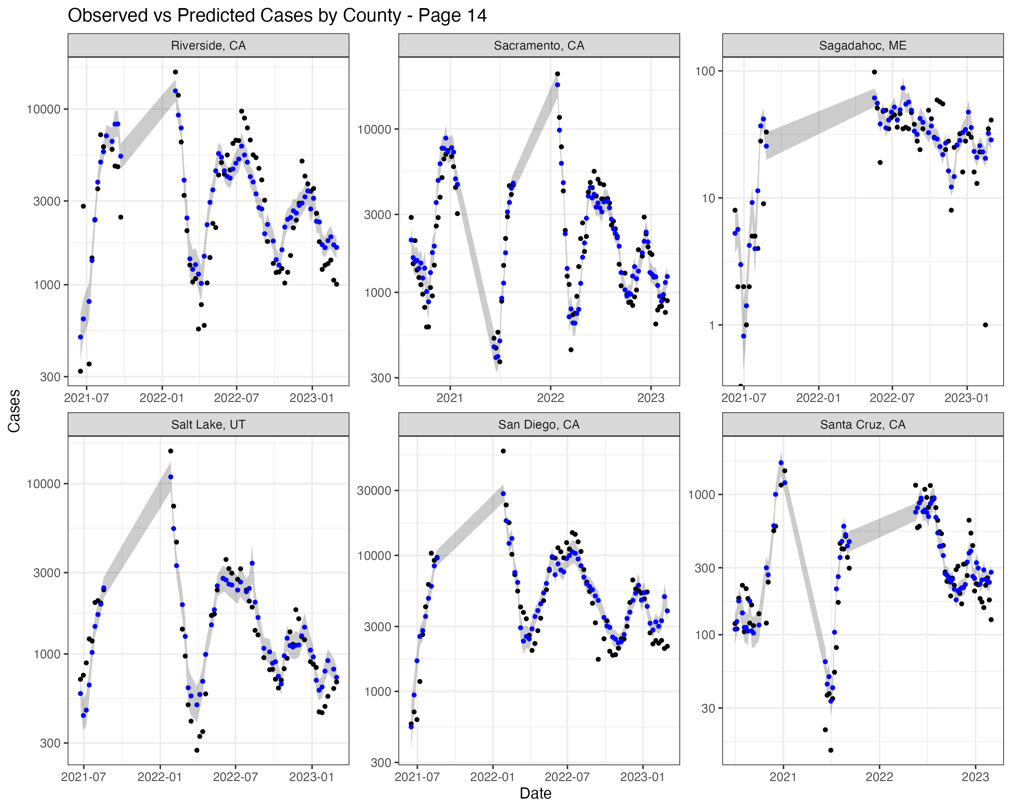

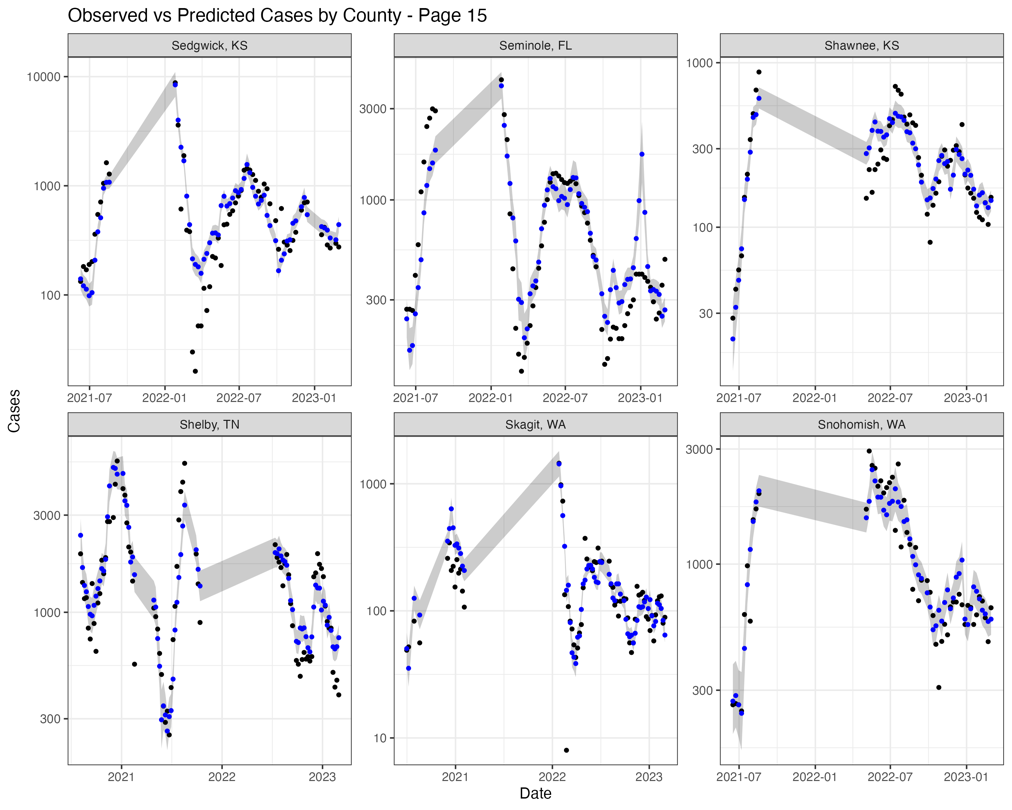

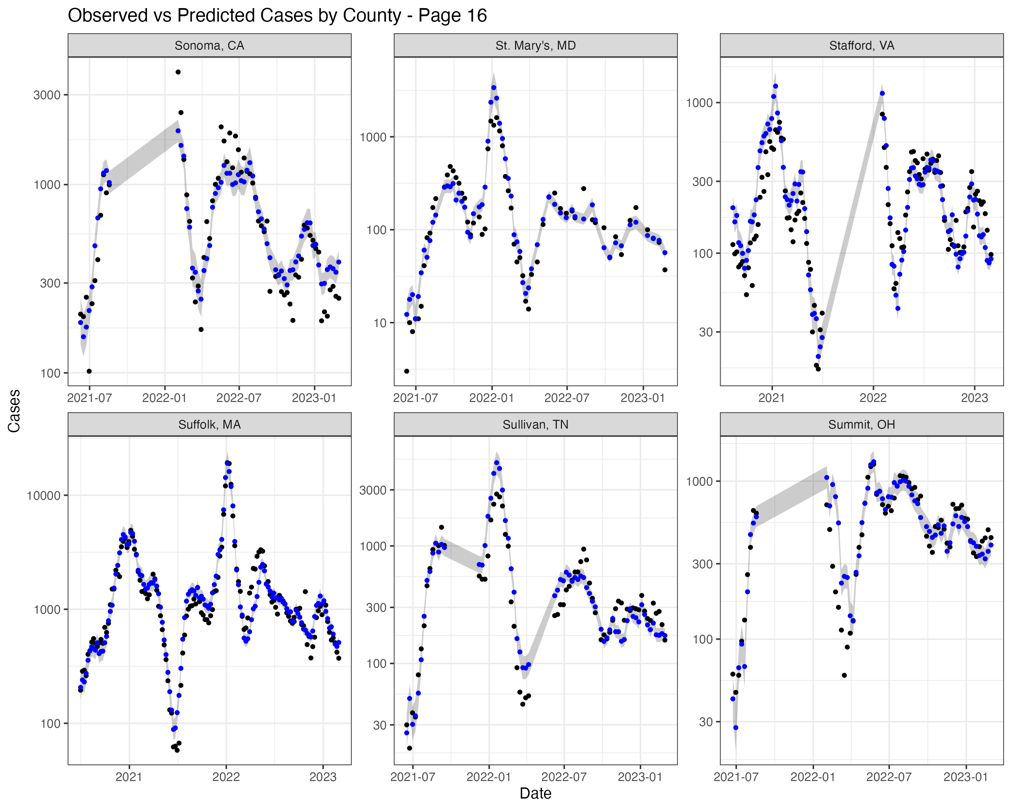

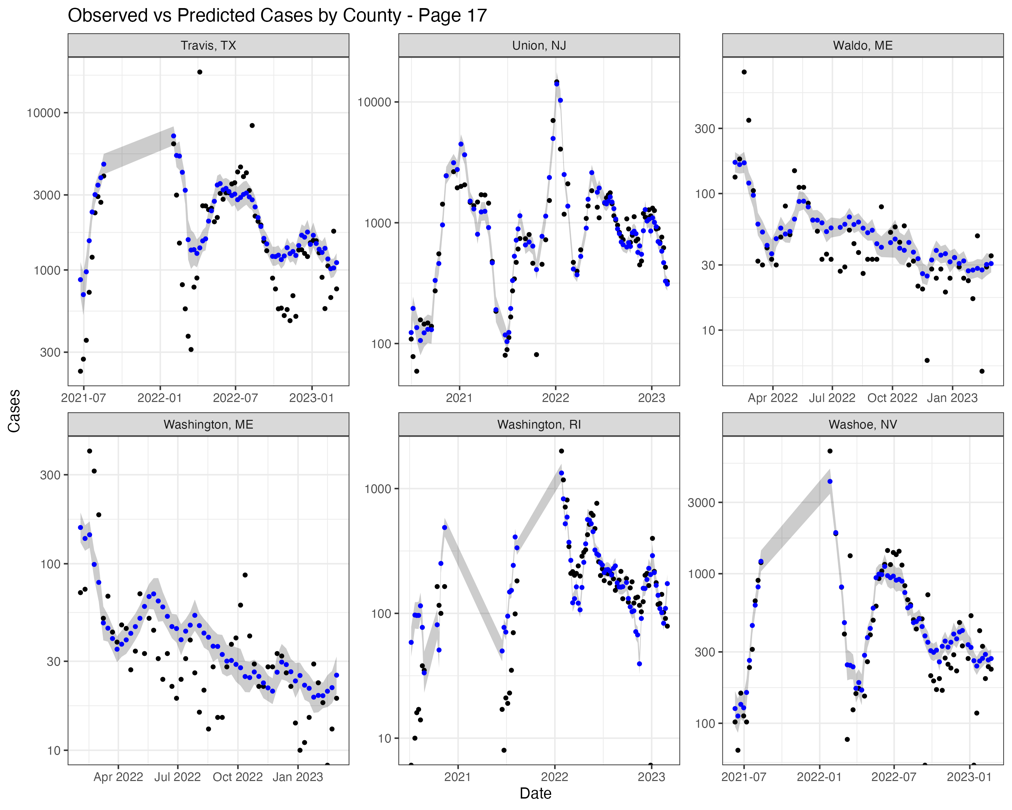

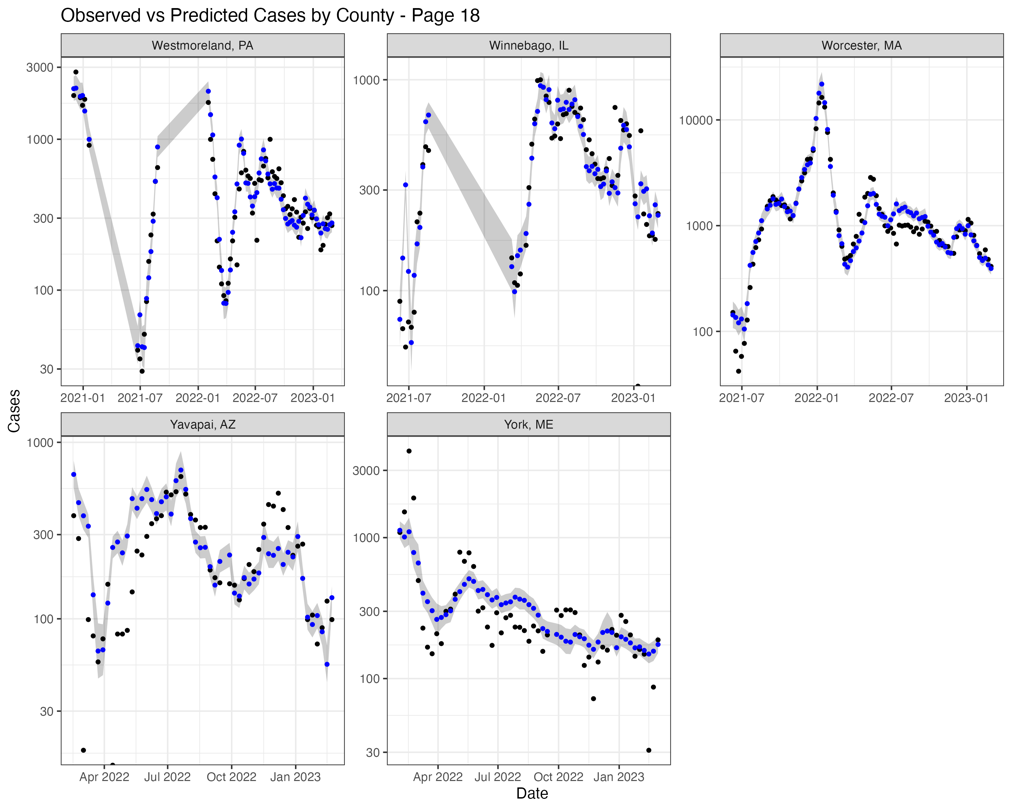


Figure S-1: The observed and predicted case counts for all 107 counties included in the analysis

## Sensitivity Analysis

We divided the dataset into three equal time periods (early, mid, and late pandemic), each spanning 11 months, and refit the model for each period. We compared the goodness-of-fit metrics, specifically the Mean Absolute Deviation (MAD), as well as key figures.

Each figure (Fig S-2 to S-6) are consistent with Figure 3 in the main paper.


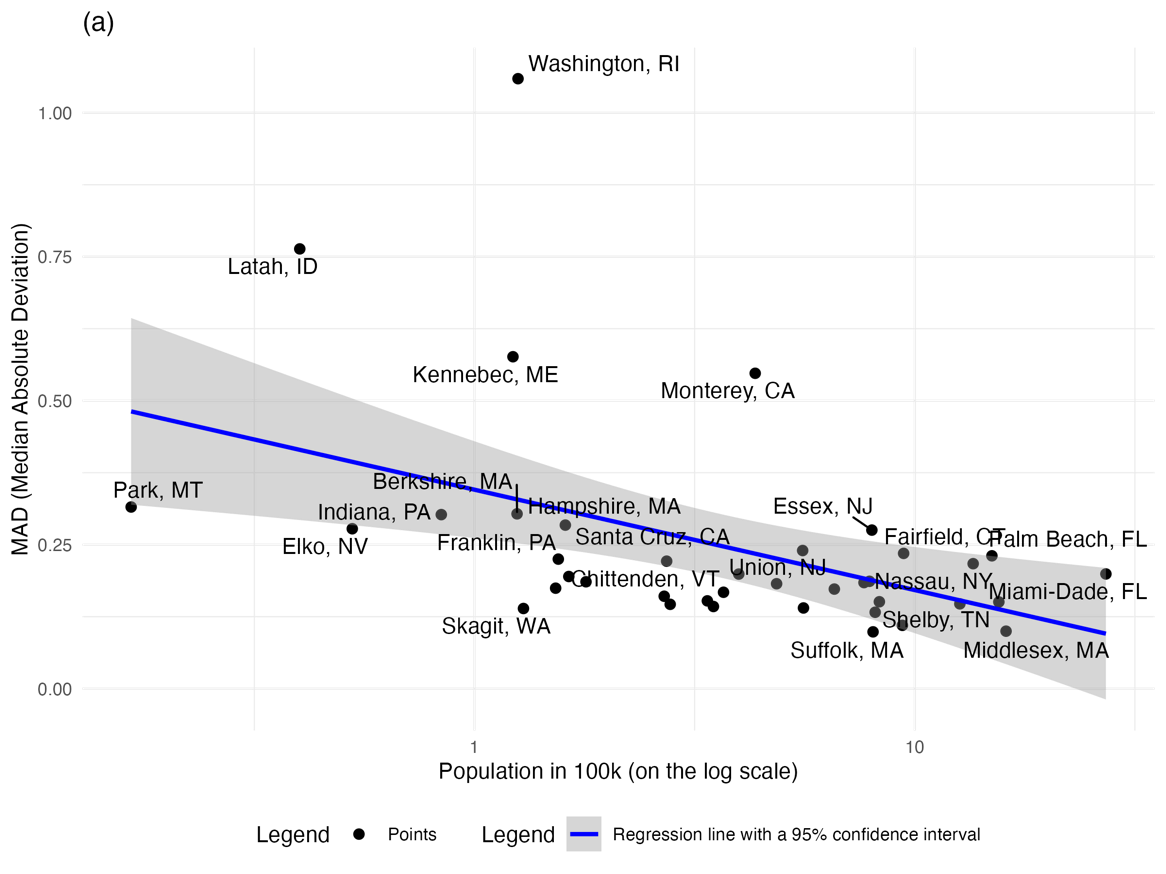


Figure S-2: This figure illustrates how the quality of model fit, measured by MAD, varies with county population size for the dataset from Period 1 (July 2020 – May 2021).


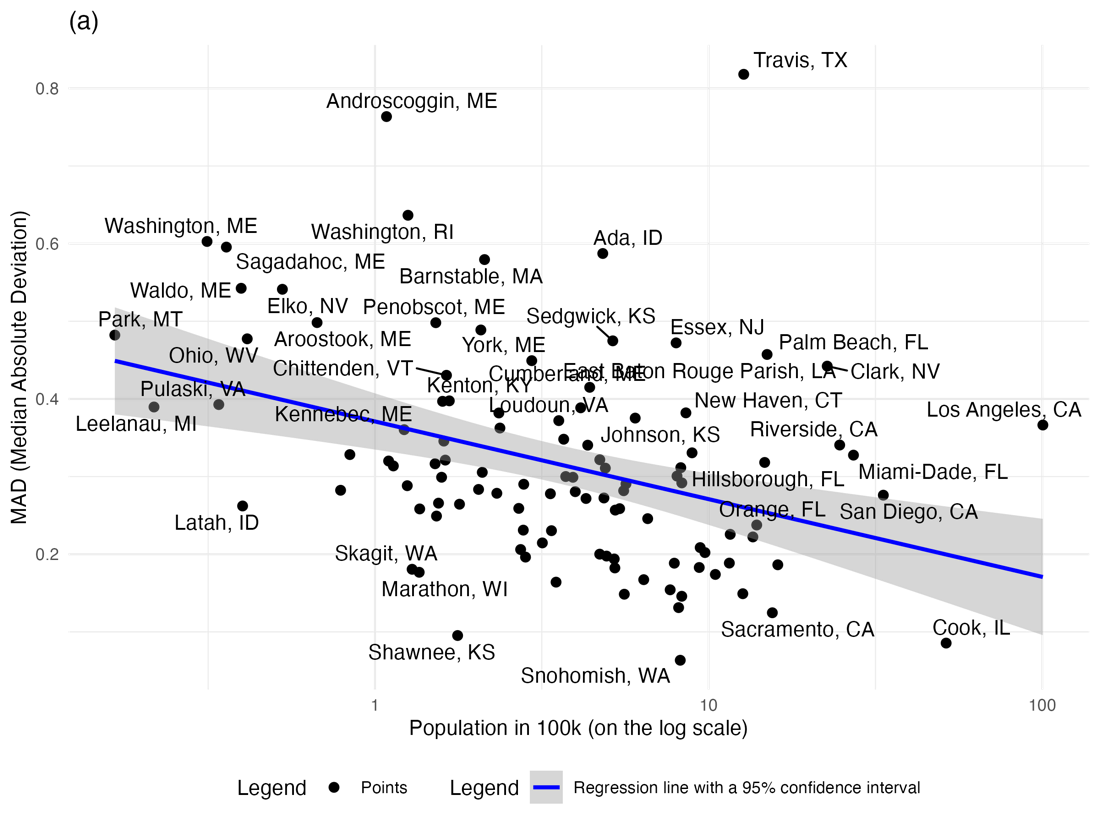


Figure S-3: This figure illustrates how the quality of model fit, measured by MAD, varies with county population size for the dataset from Period 2 (June 2021 – April 2022).


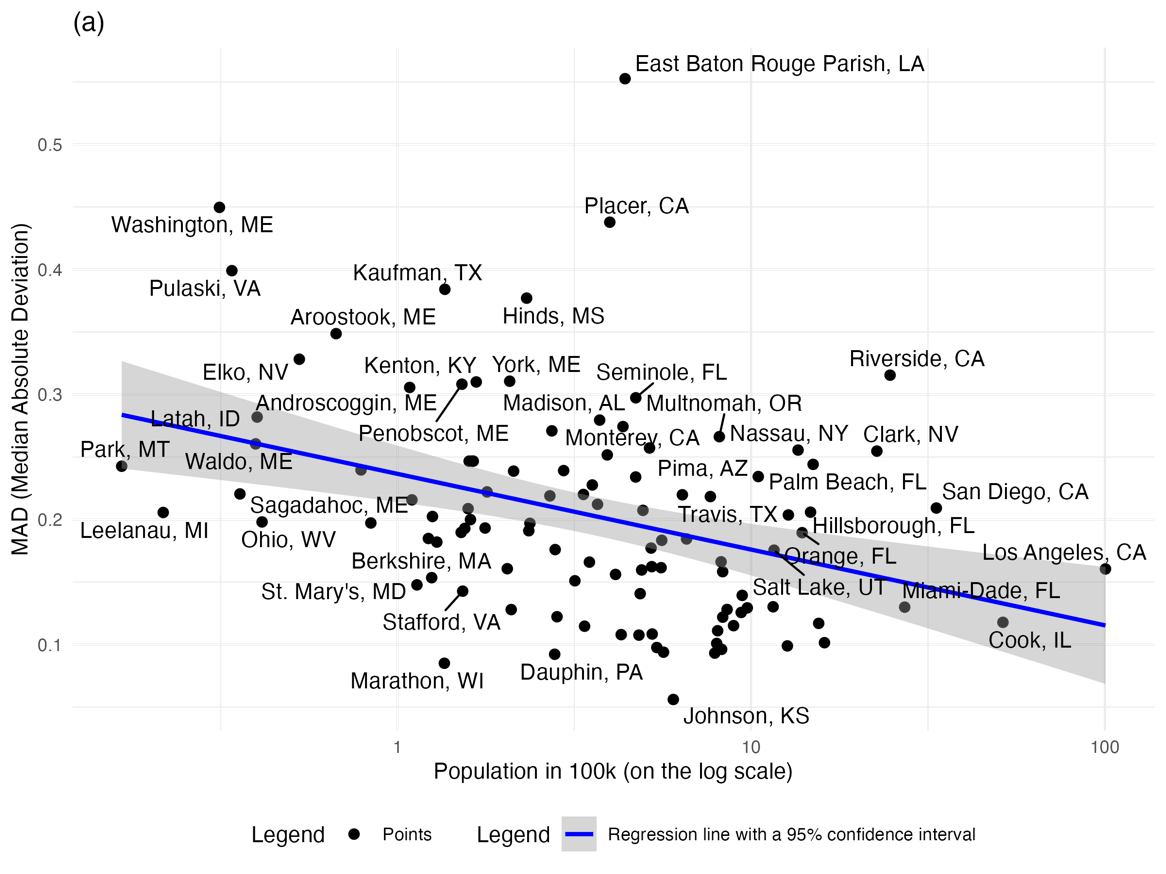


Figure S-4: This figure illustrates how the quality of model fit, measured by MAD, varies with county population size for the dataset from Period 3 (April 2022 – March 2023).


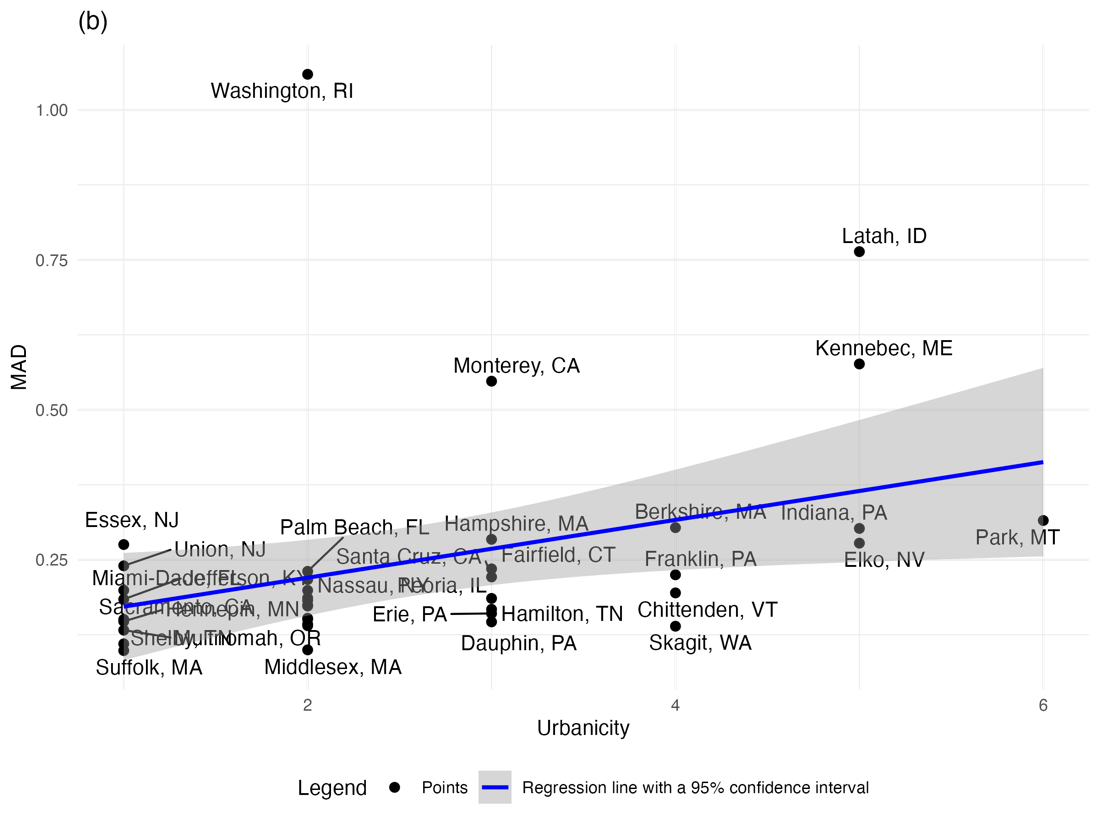


Figure S-5: This figure illustrates how the quality of model fit, measured by MAD, varies with urbanicity category for the dataset from Period 1 (July 2020 – May 2021).


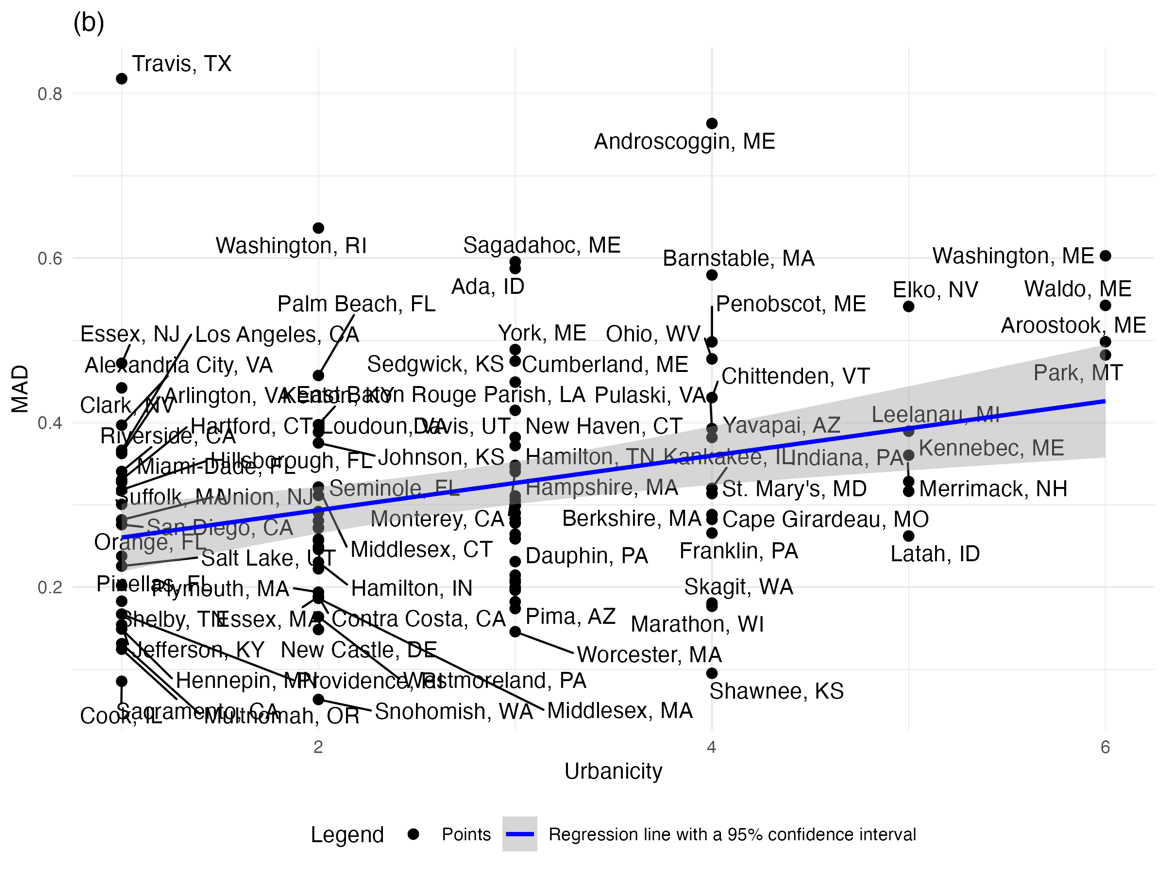


Figure S-6: This figure illustrates how the quality of model fit, measured by MAD, varies with urbanicity category for the dataset from Period 2 (June 2021 – April 2022).


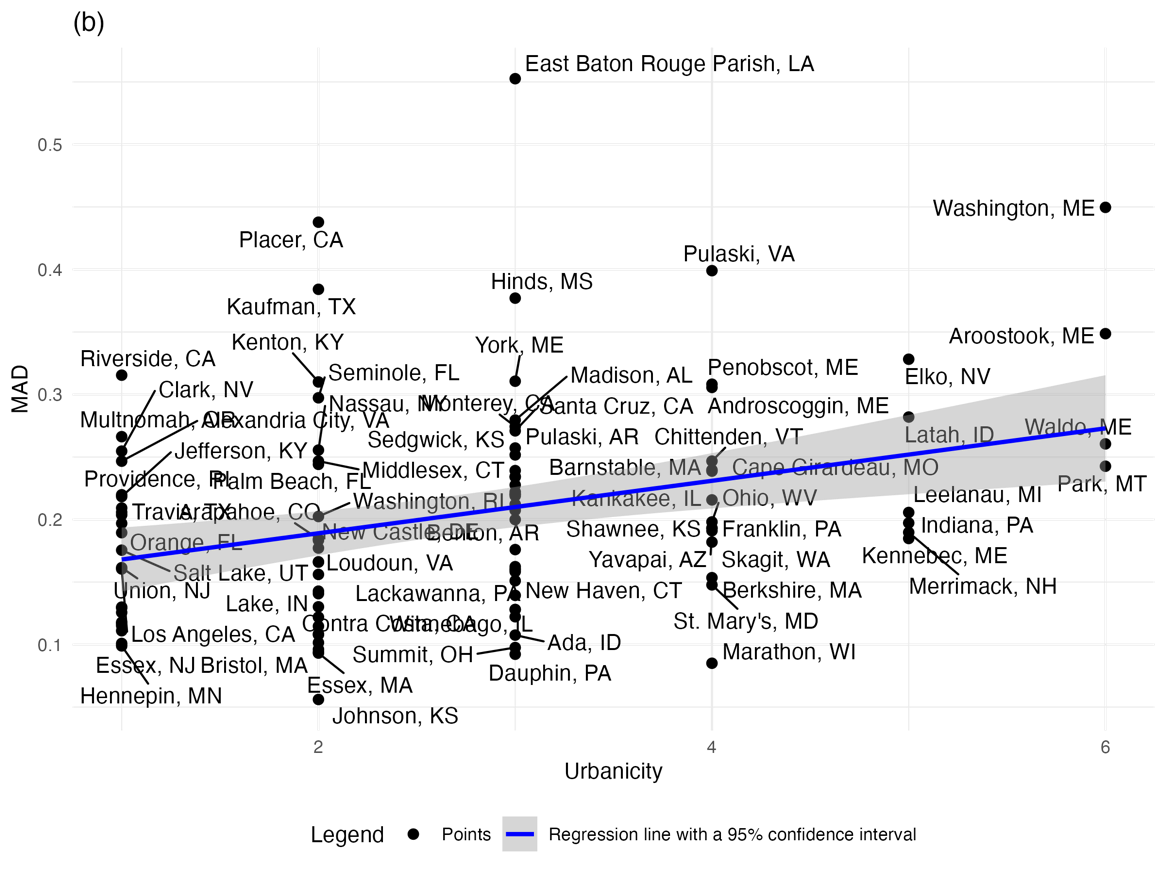


Figure S-7: This figure illustrates how the quality of model fit, measured by MAD, varies with urbanicity category for the dataset from Period 3 (April 2022 – March 2023).


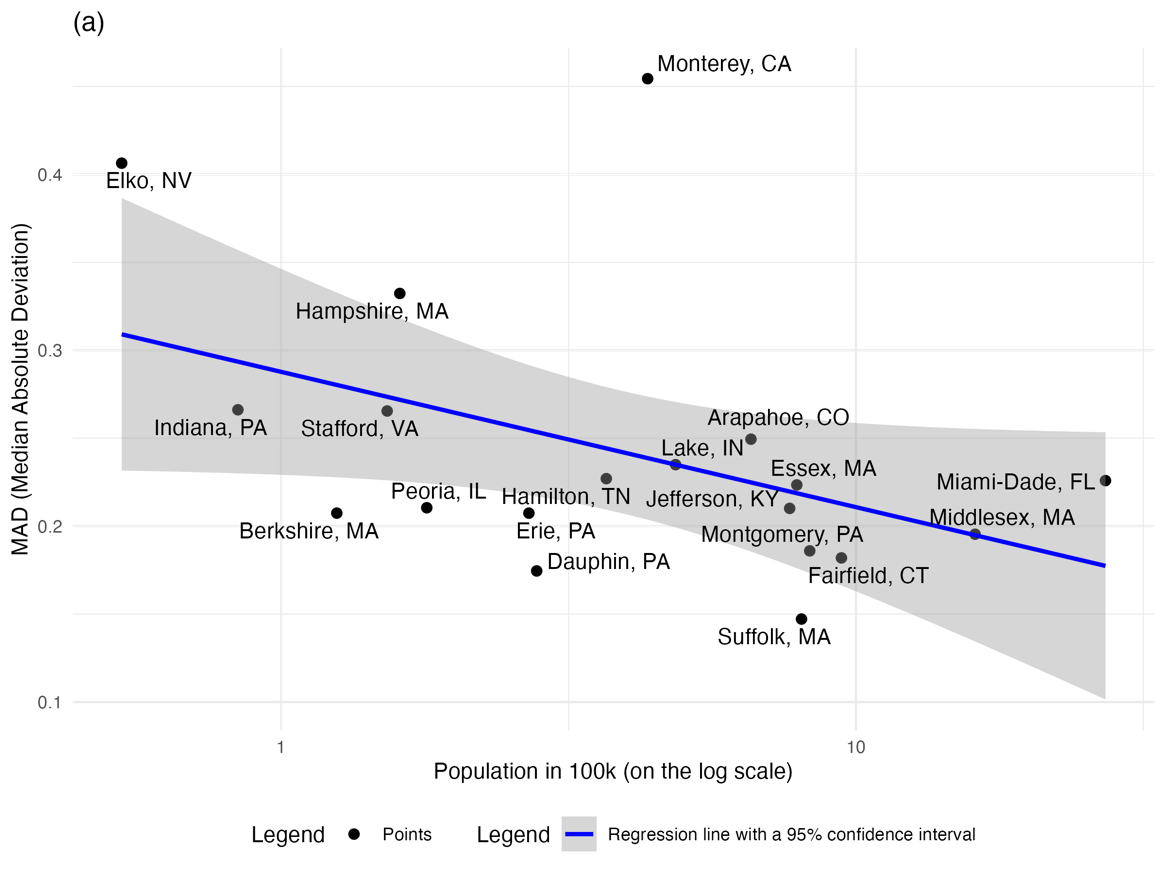


Figure S-8: This figure illustrates how the quality of model fit, measured by MAD, varies with county population size for the dataset of 19 counties that have more than 100 weeks of wastewater data.


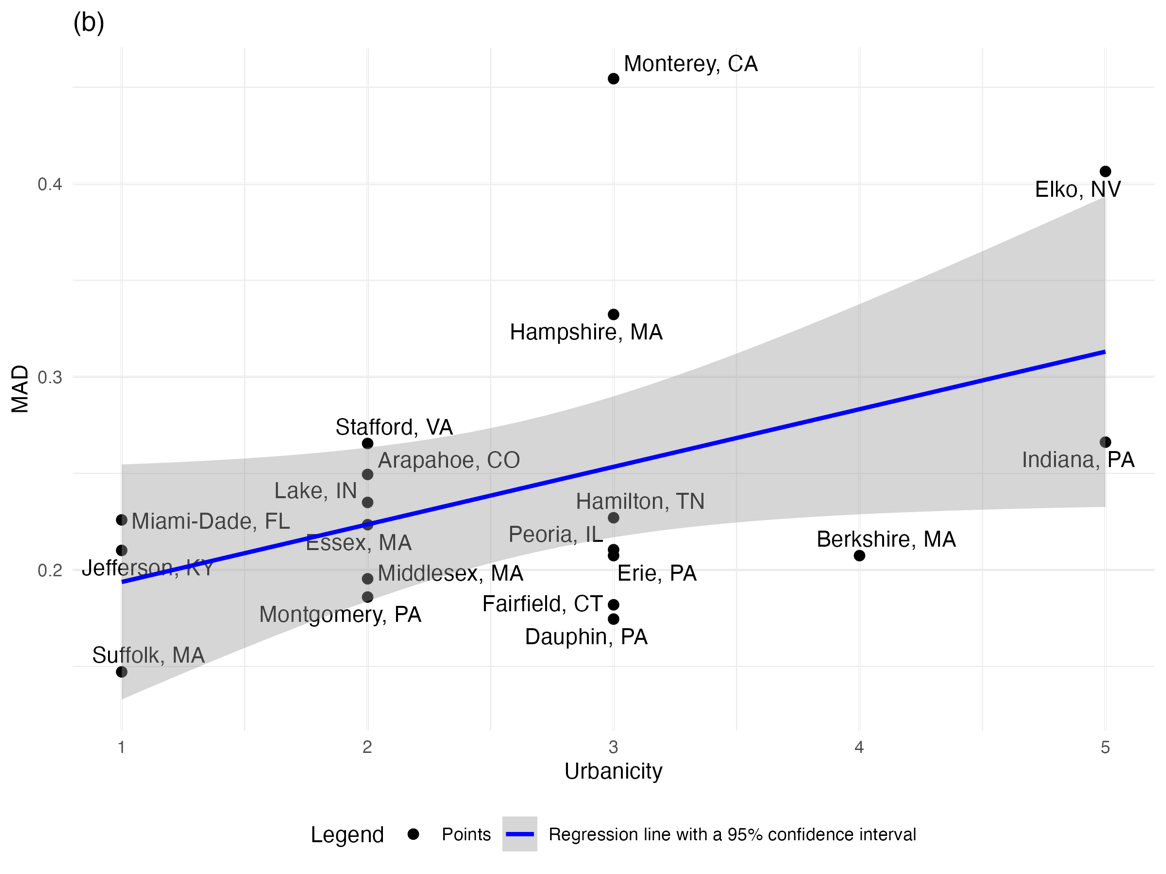


Figure S-9: This figure illustrates how the quality of model fit, measured by MAD, varies with urbanicity category for the dataset of 19 counties that have more than 100 weeks of wastewater data.
